# Vitis *vinifera* genotyping toolbox to highlight diversity and germplasm identification

**DOI:** 10.3389/fpls.2023.1139647

**Published:** 2023-04-26

**Authors:** Stylianos Tympakianakis, Emmanouil Trantas, Evangelia V. Avramidou, Filippos Ververidis

**Affiliations:** ^1^ Laboratory of Biological and Biotechnological Applications, Department of Agriculture, School of Agricultural Sciences, Hellenic Mediterranean University, Heraklion, Greece; ^2^ Institute of Agri-Food and Life Sciences, Research Center of the Hellenic Mediterranean University, Heraklion, Greece; ^3^ Institute of Mediterranean Forest Ecosystems, Hellenic Agricultural Organisation “DIMITRA“, Athens, Greece

**Keywords:** *Vitis*, biodiversity, genotyping, molecular markers, next generation sequencing, simple sequence repeats, microsatellites, single nucleotide polymorphism

## Abstract

The contribution of vine cultivation to human welfare as well as the stimulation of basic social and cultural features of civilization has been great. The wide temporal and regional distribution created a wide array of genetic variants that have been used as propagating material to promote cultivation. Information on the origin and relationships among cultivars is of great interest from a phylogenetics and biotechnology perspective. Fingerprinting and exploration of the complicated genetic background of varieties may contribute to future breeding programs. In this review, we present the most frequently used molecular markers, which have been used on *Vitis* germplasm. We discuss the scientific progress that led to the new strategies being implemented utilizing state-of-the-art next generation sequencing technologies. Additionally, we attempted to delimit the discussion on the algorithms used in phylogenetic analyses and differentiation of grape varieties. Lastly, the contribution of epigenetics is highlighted to tackle future roadmaps for breeding and exploitation of *Vitis* germplasm. The latter will remain in the top of the edge for future breeding and cultivation and the molecular tools presented herein, will serve as a reference point in the challenging years to come.

## 1 Introduction

Grapevine (*Vitis vinifera* L.) is one of the oldest and most important cultivated plants in the world (Myles et al., 2015; Laucou et al., 2018) and is believed to include between 6,000 and 10,000 cultivars worldwide. Although most of the evidence supports the appearance of domesticated vines dates eight thousand years back to the Western Asia region (McGovern, 2013), a recent article supported the hypothesis of the concurrent domestication of grapevine in Western Asia and the Caucasus (Dong et al., 2023). In the late Neolithic period, the Western Asia domesticates diffused throughout Europe, introgressed with old wild western ecotypes and diversified to give western wine ancestry.

*Vitis vinifera* L. is a dicotyledonous, multi-annual plant species that is mainly asexually propagated. Its cultivated varieties are diploid with a relatively small genome (Myles et al., 2010) that is particularly characterized by a high degree of heterozygosity (Hyma et al., 2015; Yang et al., 2016). Moreover, a plethora of *Vitis* genomes have been sequenced to-date (Jaillon et al., 2007; Velasco et al., 2007; Magris et al., 2021), providing the sequence chassis for subsequent bioinformatics analyses. Evidence shows that before its domestication, *Vitis* species were dioecious, which led to outbreeding and promoted heterozygosity. Heterozygosity was further resulted due to somatic variation (Laucou et al., 2018), which occurs in somatic cells during mitosis, usually in the form of simple nucleotide mutations that are responsible for a series of phenotypic changes such as hormone response (Vondras et al., 2019).

As a result of this centuries-long evolution, modern viticulture has been looking for suitable techniques to characterize the genetic material of the genus *Vitis* as an essential prerequisite to produce certified propagating material. In the beginning, the first attempts to discriminate cultivars have been based on phenotypic markers, though they present significant disadvantages, since they are prone to changes in environmental conditions (Nadeem et al., 2018) or are influenced by the developmental stage. Later on, biochemical markers were invented and in 1997 used to distinguish eight varieties grown is Spain (Royo et al., 1997). Nevertheless, due to the limited number and low resolution of isoenzymes the method did not get popularity (Mondini et al., 2009). In a similar concept, the analysis of complex chemical compounds, such as flavonoids, have also been used in *Vitis* but provided good discrimination power only among cultivated varieties of interspecific hybrids like those between *V. ficifolia* and *V. coignetiae* (Koyama et al., 2017). The above-mentioned techniques (morphological, biochemical) are influenced by sample manipulations (e.g. extraction process), by the developmental stage of the tissues under investigation (Nadeem et al., 2018), as well as by the environmental conditions of plant growth. Consequently, the development of genetic markers was a big step forward, mainly because they are not influenced by the environmental conditions (Royo et al., 1997; Nadeem et al., 2018), nor the developmental stage or the type of the sample, while they are abundant in the genome (Vezzulli et al., 2019).

Molecular markers used for genetic identification of *Vitis* varieties need to comply with several main characteristics: They should be polymorphic, the data produced should be publicly available to allow comparative analyses (Cabezas et al., 2011), and should produce consistent and reproducible results even after repeated propagation especially in older vine cultivars that may accumulate extensive number of mutations.

The demand for molecular markers became a necessity since the market needed tools to massively distinguish genotypes, relying on the *per se* genomic (e.g., DNA markers) or expressed genetic variability (e.g., protein markers) (Schlötterer, 2004). Although the embarking research in this field does not go back many years, its development was rapidly assisted by the parallel technological advancements concerning the invention of various next-generation sequencing approaches. PCR-based genetic analyses with molecular markers are among the most extensively used methods for the identification of *Vitis* plants or for phylogenetic studies. The most popular markers which have been used are the Simple Sequence Repeats (SSR) and the Single Nucleotide Polymorphisms (SNP) (Mondini et al., 2009). Although the use of other markers, such as Random Amplified Polymorphic DNA (RAPD) and Amplified Fragment Length Polymorphisms (AFLPs) have been initially applied, they failed to distinguish heterozygosity from homozygosity due to their dominant nature. RAPDs and AFLPs present lower reproducibility, in comparison to SSR and SNP and thus are considered less appropriate for genetic analyses in grapevine (Vezzulli et al., 2019). Furthermore, RAPDs and AFLPs are prone to subjective evaluation when the evaluator comes to the need to evaluate an increased number of bands of different intensity (Mondini et al., 2009).

The current review focus and present available data on molecular markers, epigenetic markers, DNA sequencing techniques, and algorithms that are used to discriminate cultivars and can serve as a reference point for the *Vitis* breeding programs. Despite of the plethora of publications that deal with molecular markers (Amiteye, 2021), we attempt to epigrammatically delimit the field of molecular markers with brief historical references, focusing on the technologies to come. The thorough review of the literature produced a comprehensive list of primers used in SSR analyses (**Supplementary Table 1**).

## 2 Epigenetics and *Vitis vinifera*

In the second half of the nineteenth century, grape phylloxera (*Daktulosphaira vitifoliae*), a soil-borne aphid, destroyed millions of own-rooted vineyards. To keep the cultivation alive, a rootstock survival solution had to be found. A commercial rootstock derived from a native American *Vitis* species, that co-evolved with the disease of phylloxera, displayed a higher degree of resistance (Granett et al., 1996). Studies show that the interaction of the rootstock with the graft can significantly affect epigenetic alterations and transcriptional reprogramming, and more interestingly, those changes can be heritable and may affect many other cultivation characters (Kapazoglou et al., 2021).

Epigenetics is the study of the heritable changes in gene expression that occur without changes in the underlying DNA sequence. In plants, such modifications can influence phenotypic plasticity, which is the ability of a plant to change its phenotype in response to changes in environmental conditions. Epigenetic modifications include the DNA methylation or demethylation, histone modification, the remodeling of chromatin organization, and the action of specific classes of small RNAs and long non-coding RNA (Fortes & Gallusci, 2017). Recently, Kapazoglou et al. (2021), reviewed these mechanisms and associated them with grafting and rootstock interactions. Because of the existence of sufficient phenotypic variation within clones of the same variety that cannot be attributed to DNA differences, the grapevine could serve as a plant model for studying epigenetic mechanisms in perennial woody plants (Fortes & Gallusci, 2017).

Several studies discuss the role of DNA methylation in phenotypic plasticity. For example, it was demonstrated that phenotypic differences observed in Malbec clones grown under two opposing environmental conditions (Varela et al., 2021) were due to the different epigenetic profile of the clones. In another study, Almada et al. (2011), found evidence that epigenetic repressor-like genes were differentially regulated during grapevine development. Tyunin et al. (2013) showed that the methylation on cytosine DNA residues is crucial for the regulation of the resveratrol biosynthetic pathway. Xie et al. (2017), observed that changes in DNA methylation between vineyards or vineyard sub-regions cultivated with the same Barossa variety were caused by both geographic location and, to a lesser extent, by pruning strategy. Ocaña et al. (2013), discovered stable methylation-sensitive amplification polymorphism (MSAP) markers that effectively distinguished 40 clones of Pinot noir cultivar, demonstrating the use of this type of methylation markers in genotyping. Similarly, Schellenbaum et al. (2008), successfully exploited MSAP markers to distinguish somaclones when SSR and AFLP failed. Interestingly, in another study from Baránek et al. (2015), it was observed stable mitotically inherited methylation diversity, that remained after plants were stressed *in-vitro*. It was also shown that the atypical phenological traits which were observed after somatic embryogenesis, on Sultana variety, were associated with epigenetic factors (Franks et al., 1998). In another study by Xie et al. (2017), it was reported that the global DNA methylation patterns could explain the terroir influence in grapevine, and that the epigenetic differences, highlighted by MSAP, in samples from 22 vineyards and six sub-regions of the Barossa Wine Zone, grouped vineyards based on their geographic location. In contrast, Dal Santo et al. (2018), found that there was not any fluctuation in DNA methylation profile or in gene expression in grapes and plant genotype was the main factor of the methylation variance between samples.

Recently, Lewsey et al. (2016), showed that mobile RNA may regulate the DNA methylation landscape genome wide, that also play important role in pathogen defense mechanism in crops. Furthermore, it was showed that for kaolin particle film treated plants, methylation of DNA was decreased in leaves, a response that could be associated with the harsh environmental conditions (Bernardo et al., 2017). Fabres et al. (2017), also pinpoint the important factor of combination of epigenomics when integrating omics data as epigenetic mechanisms can play a major role between the genome and the environment in *Vitis* germplasm. Therefore, the environment can have a long lasting phenotypic effect on *Vitis* through epigenetic modifications, even on the same genomic background. These modifications may work as intermediaries between environmental variation and the plant genome, and in this way, may potentially contribute to plant phenotypic plasticity.

## 3 Most commonly used genetic markers: simple sequence repeats

Simple Sequence Repeats or Microsatellite Markers are a category of abundant Short Tandem Repeats (STRs) scattered throughout the genome that make up genomic repetitive regions (Agarwal et al., 2008; Kantartzi, 2013; Tello and Forneck, 2019). They usually concern 5-50 “monotonous” repetitions of the type (GA)n, or (GATA)n or similar minute sequence repeat. Indicatively, Velasco et al. (2007), estimated the number of SSR in the genome of a highly heterozygous genome of *Vitis vinifera*, at about 89.000. They are considered as evolutionarily neutral DNA markers (Li et al., 2002) and tend to mutate at rates between 10^-3^ and 10^-6^ (Gemayel et al., 2012) that may reach the frequency rate of 10^-9^ (Madesis et al., 2013) per cellular generation in certain circumstances. The latter means that it is up to ten orders of magnitude larger than point mutations (Gemayel et al., 2012) and thus, may be used for linkage mapping studies, association studies, or identification of organisms.

Sweet et al. (2012), summarized the three proposed theories that may explain the existence of SSRs. Firstly, they probably constitute a kind of “junk DNA” that is present inside the genome having a rather neutral role. A second interpretation states that their presence ensures a source of genetic variation that helps organisms to adapt to different environments, and thirdly, they may possess a possible regulatory role in gene expression. Nevertheless, the “junk DNA” term has been degraded since there are evidence that SSR present actions on chromatin organization, regulation of gene activity, recombination, DNA replication, cell cycle, or mismatch repair system (Li et al., 2002).

SSR markers have been frequently used for genetic analyses of *Vitis* individuals as it has been shown by the increased number of publications (Bacu et al., 2015; Bitz et al., 2015; Dallakyan et al., 2015; Ferreira et al., 2015; Maletic et al., 2015; Khadivi et al., 2017; Popescu et al., 2017; Labagnara et al., 2018; Karatas, 2019). They are frequently used to analyze the grapevine germplasm due to their multiallelic nature and may be used to solve homonym and synonym issues, to determine genetic variability among *Vitis vinifera* cultivars as well as to establish pedigree analysis (De Lorenzis et al., 2015b). A broad collection of the SSR markers showed in the literature along with a detailed compilation of related data, are shown in **Supplementary Table 1**.

Each SSR primer pair has a different ability to detect polymorphism within a population. Two parameters linked with diversity measures are the Heterozygosity (H) (Nei and Roychoudhury, 1974; Botstein et al., 1980; Nagy et al., 2012) and the Probability of Identity (Pi), a measure of the probability that two randomly chosen individuals in a population have identical genotypes (Emanuelli et al., 2013). Polymorphic markers are considered those that present at least two different alleles, whose frequency is lower than 100% (Serrote et al., 2020). The bigger the PIC number is, the greater the variety of alleles in the studied place will be. According to **Supplementary Table 1** the SSR loci proposed by the OIV are very informative and are suitably used in technical protocols for genetic analyses. On the opposite side, SNP are also useful for management of *Vitis* germplasm. The latter are getting more attention although SSR are more informative than SNP mainly because of their abundance. In the same work they concluded that one SSR locus provides the same information as 2,5 SNP loci. Cabezas et al. (2011), developed a protocol for the detection of 48 SNP that provided increased discrimination power partially due to even genome distribution (2-3 markers per chromosome). This protocol was successfully used for the genetic analyses of 200 *Vitis* varieties.

Similarly, the selection and cultivation of clones provides an exceptional opportunity to modern viticulture since they contribute to the diversity of germplasm and subsequently to breeding and the development of new varieties. The differentiation of *Vitis* clones is not always straight forward. When different clonal genotypes were analyzed with a small number of markers, differentiation was unsuccessful (Vignani et al., 1996; Silvestroni et al., 1997). This agrees with the findings who reported that SSR are inappropriate for *Vitis* clone identification especially when they are closely related. One of the first successful attempts is that of Jahnke et al. (2011), who claimed to have successfully differentiated varieties and clones of various types of Pinot cultivars with SSR markers. However, the success could be due to the wide geographical distribution of the clones.

There is a plethora of publications where SSR markers are utilized for either identification or phylogenetic analyses of *Vitis* cultivars. Recently, concluded that the utilization of 20 SSR markers is sufficient to distinguish existing cultivars or to solve synonym and homonym issues (Stavrakaki et al., 2020). A common criterion for the eligibility and appropriateness of a marker is the degree of polymorphism that can highlight differences between cultivars and ideally clones (Jahnke et al., 2011). The International Organisation of Vine and Wine (OIV) attempted to harmonize the international criteria for grapevine cultivar identification, to provide a protocol for official recognition and registration of a variety (OIV, 2019).

Nowadays, the basis for differentiation between cultivars is the utilization of the nine OIV SSR loci (OIV, 2019; **Supplementary Table 1**), as they have been proposed to offer the necessary resolution, although less than nine have been also used (Stajner et al., 2015). Nevertheless, there are groups that have used custom combinations of SSR markers to support their claims (**Supplementary Table 1**). The utilization of SSR markers has been expanded to include wild *Vitis* accessions like *V. sylvestris* or *V. sativa* (Zdunic et al., 2017). The first six OIV markers (VVS2, VVMD5, VVMD7, VVMD27, VrZAG62, VrZAG79) were evaluated under the EU Project GENRES081 (This and Dettweiler, 2003) and have been proved suitable for grapevine variety characterization due to their ability to produce high degree of allelic polymorphisms (13-23 alleles per locus) and establishing high discriminatory power (Jung et al., 2008). The remaining three (VVMD32, VVMD25, VVMD28) were evaluated under the program GrapeGen6 (GrapeGen6, 2012) and produced highly frequent alleles among 2901 Vitis accessions. However, argued that at least twenty SSR markers are needed to efficiently distinguish/identify each vine variety and proposed the set consisting of VVS2, VVMD5, VVMD7, VVMD21, VVMD24, VVMD25, VVMD27, VVMD28, VVMD32, VMC4f3, VVIn73, VVIp31, VVIp60, VVIQ52, VVIV37, VVIV67, VVIn16, VVIh54, and VMC1b11 (**Supplementary Table 1**).

While SSR are highly polymorphic and widely distributed throughout the genome, theoretically, they could be used to assess genetic diversity even among individuals with subtle genomic differences. Despite the widespread use to identify genomic diversity among varieties, they are incapable to resolve close genetic differences. In a study by Imazio et al. (2002), they used three different types of markers, SSR among them, to differentiate between 24 clones of the grape variety Traminer. They found that SSRs were not a powerful tool for clonal distinction and that clonal identification could be greatly improved using AFLP or MSAP. In a similar study, concluded that although SSR markers are adequate for cultivar identification, they are definitely not well suitable for clonal identification.

Among the advantages of SSR is the use of species-specific primers, and the presence of increased variability and reproducibility (Núñez et al., 2004). Data from SSR analyses may be stored in publicly available databases. Since they exert co-dominancy (Núñez et al., 2004; Meudt and Clarke, 2007; Nadeem et al., 2018), SSRs can be used to differentiate homozygous from heterozygous individuals (see detailed data in **Supplementary Table 1**). Their presence in expressed sequence tags (EST) allows not only the discrimination of *Vitis* cultivars but also to point-out the association with genes of interest for the creation of genetic maps (Núñez et al., 2004).

SSRs are abundant covering the entire genome (Qin et al., 2015) and they appear to be an important tool for almost any problem requiring Mendelian genetic markers. The results from SSR-PCR analyses are easily assessed, consistently scored and thus, they can be exchanged between different research evaluations. Furthermore, their detection is based on simple PCR-amplifications, offering great practical advantage over other methodologies (AFLP, RFLP, VNTR, etc.). Even poorly preserved specimens may be valuable sources of material, since SSR analysis may be applied to samples with degraded DNA (Queller et al., 1993; Qin et al., 2015).

In contrast, the SSRs’ main drawbacks are the difficulty to detect new SSR loci, the laborious nature of this process until they reach to a detection protocol, and eventually the cost (Madesis et al., 2013). The development of NGS technology will serve as a helpful alternative. Nevertheless, once found, microsatellites are easily utilized to generate abundant and unambiguous genetic data that are likely to become an indispensable part of many evolutionary studies (Queller et al., 1993). Although, in general, SSRs are reproducible, technical difficulties may arise when amplifying SSR loci, particularly in the detected length of amplicons that differ in only one or two base pairs (Madesis et al., 2013; Laucou et al., 2018).

## 4 Single-nucleotide polymorphisms: simple and abundant

Single-nucleotide polymorphisms or SNPs are currently the most widely used markers mainly because of their wide distribution in the genome (Broccanello et al., 2018). They are single base pair positions in genomic DNA, where the allele with the lowest incidence is found in an abundance of at least 1% or more (Jehan and Lakhanpaul, 2006). SNPs are considered the basis of phenotyping diversity as they can affect the gene or protein expression. It is estimated that they account for more than 80 percent of all polymorphisms observed in an organism (Komar, 2009). Among the main characteristics of SNP are a) the very large number of loci can be surveyed simultaneously, especially coupled with next-generation sequencing platforms, and b) the relative ease of scoring and analysis of data.

SNP may have up to four alleles per locus but usually only two are present due to low mutation rates (Glaubitz et al., 2003). Although SNP are less informative than microsatellites, they are abundant in the genomes and tens of thousands of SNPs may be detected quickly and cheaply *via* microarrays (Marrano et al., 2017; Laucou et al., 2018) or may be exploited by High-Throughput Sequencing (HTS) automation (Salmaso et al., 2004; Mammadov et al., 2012; Yang et al., 2016; Marrano et al., 2017). Thus, they show a higher ability to detect genetic variability due to abundance and whole-genome localization. Moreover, they provide better resolution to elucidate the relationships between the samples at the population level, as well as they can be applied for parentage analysis. It is worth noting that SNP profiles from one laboratory can be easily compared with those generated from other laboratories (De Lorenzis et al., 2015a).

They also have co-dominant nature (Salmaso et al., 2004; Zyprian et al., 2015) and may be used for association studies between alleles and phenotypes (Rafalski, 2002). Furthermore, SNP present higher degree of reproducibility and reliability among different laboratories (Vezzulli et al., 2008; Laucou et al., 2018). SNPs are more advantageous over SSR mainly due to their abundance in the genome and thus are suitable for parentage or kinship analysis (Laucou et al., 2018). It has been shown that SNP polymorphisms are very useful to understand population structure, perform clone identification, conduct segregation and phylogenetic analyses (Nicolas et al., 2016; Li et al., 2019), assist plant breeding, or manage genetic resources in germplasm collections (Kumar et al., 2012a). Also, SNPs constitute the basic markers in Genome Wide Association studies (GWA), while they allow high-throughput automation (Marrano et al., 2017). A summary of the main properties (advantage*s* and disadvantages) of SSR and SNP used in genetic analyses of *Vitis* cultivars is given in **Table 1**.

**Table 1:** Basic features of SSR and SNP molecular markers used for the identification of *Vitis* cultivars.

Dominant Marker/ Codominant Marker	SSR	Codominant Marker (Nadeem et al., 2018)
	SNP	Codominant Marker (Nadeem et al., 2018)
Biallelic/Multiallelic	SSR	Multiallelic (Cabezas et al., 2011)
	SNP	Biallelic (Cabezas et al., 2011)
Discrimination ability	SSR	○ Difficulty separating electrophoretic bands that differ in only one or two base pairs (Madesis et al., 2013) z Due to their multiallelic nature they show increased genetic information compared to SNP (Kantartzi, 2013, Zyprian et al., 2015) ○ Miscalling difficulties when di-nucleotide repeats are utilized ○ Frequent addition of an Adenine nucleotide by DNA polymerases (Cabezas et al., 2011)
	SNP	○ SNP show a higher ability to distinguish especially between close relatives than SSR. Due to their biallelic nature, SNP show reduced genetic information compared to SSR but this disadvantage is offset by their abundance in the grapevine genome (Zyprian et al., 2015) ○ In the kinship analysis a main advantage of SNP markers (*in relation to SSR*), is that due to the increased number of available SNP markers, lead to an increased LOD score and therefore to a greater chance of discrimination in first degree relatives with second degree relationships (full-sibling vs. second-degree) (Laucou et al., 2018)
Monolocus marker/ Multilocus marker	SSR	Monolocus marker (Khlestkina and Salina, 2006).
	SNP	Monolocus marker (Khlestkina and Salina, 2006)
Competent to sequencing	SSR	Yes (Nadeem et al., 2018)
	SNP	Yes (Nadeem et al., 2018)
Level of Detection of Polymorphisms in the Grapevine	SSR	○ In the primer recognition site, some individuals will have only one allele amplified or fail to amplify the genetic loci ○ In different species, alleles of the same size may be created but not identical [homoplasy (Sweet et al., 2012)] ○ The mutation rate of SSR loci varies at a rate between of 10^-9^ to 10^-3^ per cell generation (Gemayel et al., 2012; Madesis et al., 2013) ○ Average PIC (Polymorphism Information Content or Gene Diversity Index): 0.63 ± 0.15 (Khlestkina and Salina, 2006)
	SNP	○ They show reduced genetic information compared to SSR, ○ Average PIC (Polymorphism Information Content or Gene Diversity Index): 0.27 ± 0.23 (Khlestkina and Salina, 2006)
Abundance	SSR	○ The number of SSR primers is limited and it is difficult to develop new primers, as many sequences need to be cloned and only a small number of them will be useful for developing SSR markers. In addition, only some of these markers will provide useful information, especially for species with large genomes (Kantartzi, 2013, Madesis et al., 2013)
	SNP	○ Abundant (Marrano et al., 2017)
Transferability	SSR	○ Not as useful as SNP in improving the traceability and identification of germplasm (Marrano et al., 2017)
	SNP	○ Existence of doubts about the transferability between varieties, hybrids or clones (Zyprian et al., 2015)

## 5 Other type of markers

In addition to the use of SSRs and SNPs, other types of molecular markers have been applied (ISSR/Inter Simple Sequence Repeats, AFLPs, M-AFLPs) but are generally laborious, costly, time consuming and difficult to apply in low-skilled laboratories. However, the development of Next Generation Sequencing technologies may assist towards the reliable detection of grape genetic diversity even among clones of the same variety.

## 6 Algorithms and software packages to infer phylogenetic relationships among cultivars

Algorithms are utilized to infer the evolutionary connections between a group of organisms or sequences based on molecular or morphological data. The outcome of a phylogenetic analysis is a tree, which displays the putative evolutionary connections among the sequences. The construction of the tree is an essential step in phylogenesis because it is the process of finding the tree that best explains the data according to a specific criterion, such as maximum likelihood or maximum parsimony, that reflects the most likely evolutionary history of the sequences or organisms under study. This can be performed using different algorithms and models, depending on the nature of the data and the assumptions underlying the analysis.

Regardless of the methodology that is being utilized to reveal grapevine genetic or genomic variability (SSR, SNPs, etc.), the selection of the algorithm or method to infer the clustering analysis of populations and cultivars, is critical. Algorithms are, essentially, the equations that various statistical software use to quantify variability and finally present it as a branched tree (dendrogram or phenogram as in Lefort and Roubelakis-Angelakis (2001); Karataş et al. (2007); Alifragkis et al. (2015)). In the case of molecular marker analysis there is a plethora of different algorithms or methods that may be utilized and can be classified into three main groups, distance methods, parsimony methods and maximum likelihood methods (Nei and Kumar, 2000; Ochieng et al., 2007).

In the distance methods the evolutionary distances (or pairwise differences) and the construction of phylogenetic trees are put together by grouping individuals (or genotypes) that are most similar (Uncu et al., 2015). Examples of distance-based methods include Neighbor Joining (Saitou and Nei, 1987; Pardi and Gascuel, 2012; Doulati-Baneh et al., 2013) and Unweighted Pair Group Method with Arithmetic Mean (UPGMA, Sneath and Sokal, 1973). Parsimony-based methods identify the tree topology that requires the fewest number of evolutionary changes (such as substitutions, insertions, and deletions) to explain the observed variation in the sequences analyzed. The method assumes that the evolutionary changes occur independently and equally likely at each site. Maximum likelihood methods estimate the likelihood of observing data under different models of evolution and tree topologies. The likelihoods are then used to identify the tree topology that is most likely to have generated the observed data. Maximum likelihood methods typically use complex models that account for variations in the rates and patterns of evolutionary changes across sites and branches of the tree.

Abovementioned methods can be a useful and informative approach for reconstructing the evolutionary history in populations and identifying patterns of genetic variation among individuals or groups by the application of any type of molecular data, including SSR (Uncu et al., 2015). Each method has its own advantages and disadvantages and is suited for different types of data and research questions. Distance methods are computationally efficient and can be used for large datasets, but they do not account for variations in the rates and patterns of evolutionary changes. Parsimony methods are simple and intuitive, but they may not be the most accurate method when the evolutionary changes are complex and varied. Maximum likelihood methods are more complex and computationally demanding, but they can account for complex evolutionary models and provide more accurate estimates of the phylogeny (Nei and Kumar, 2000; Uncu et al., 2015).

Besides the different algorithms that have developed to infer phylogeny, there are also different software packages that may assist the calculation of the genetic distances or perform clustering analyses based on the specified algorithms. A commonly used criterion or clustering algorithm is the UPGMA. The UPGMA, a simple bottom-up hierarchical clustering method, was utilized by Zoghlami et al. (2013), to evaluate the genetic structure of a wild *Vitis* population. Data were fed to the Darwin software program with a matrix of pairwise FSt values. Darwin was also used by Goryslavets et al. (2015), to study the diversity of ancient grape cultivars of the Crimea region. They also utilized the weighted version of Neighbor-Joining method that was similarly used by employing the simple matching distances (Bowcock et al., 1994). Moreover, Moreno-Sanz et al. (2011), also utilized UPGMA, based on Jaccard’s similarity coefficient and cophenetic correlation coefficient but instead worked with WinBoot software. Additionally, they performed a Bootstrap analysis with 1.000 replicates. The latter is critical since it provides a way to estimate the standard error as well as the confidence intervals of the analyses but unfortunately is not widely used. Martínez et al. (2006), highlighted the diversity in Peruvian and Argentinean genotypes employing the NTSYSpc (Rohlf, 2008), similarly to De Lorenzis et al. (2013), who studied the diversity of a group of *Vitis* genotypes from Italy, Slovenia and Ionian islands along with the package PHYLIP (Felsenstein, 2004). In general, NTSYSpc is a popular software package for analyzing multivariate data. It has been used in several studies on grape inter-varietal genetic diversity (Santiago et al., 2005; Meneghetti et al., 2009; Castro et al., 2011), though it was loaded with different types of similarity matrices (allelic data as squared distance matrices, simple matching coefficient, proportion of shared alleles or DICE coefficient).

MEGA software (Tamura et al., 2021) is another widely used software package for molecular evolutionary analyses that was used to study the diversity of selected grape genotypes from the island of Crete and from Iran (Doulati-Baneh et al., 2013), based in SSR markers. In a similar work, utilized SSR markers and then applied the Jaccard index (Jaccard, 1908) to highlight the genetic similarity, which was used for cluster analysis to discriminate Hungarian *Vitis* varieties. This is also known as nearest-neighbor approach.

It is evident that there is a plethora of algorithms and software packages to study phylogenesis and interpret phylogenetic relationships between cultivars. Therefore, it is important to carefully consider the strengths and limitations of each method when selecting an appropriate approach for analyzing data to adequately compare *Vitis* varieties. Until today, there is no formula or recommendation about the optimum algorithm that can be used to adequately compare *Vitis* cultivated material. The choice of method depends on the specific research question, the characteristics of the data being analyzed, and the assumptions that are appropriate for the biological system under study. Often, it is advantageous to compare the results of many methods to ensure that the inferred tree is robust and not disproportionately influenced by the selected method.

## 7 High-Throughput strategies for Genotyping of *Vitis* cultivars and clones

### 7.1 Utilization of immobilized oligonucleotides in microarray formats

Recently, diversity analysis of grapes was exploited by the use of microarrays bearing immobilized oligonucleotide strings representing a very large number of SNP from various species. Myles et al. (2010) was the first to develop an array consisting of 9.000 SNP (Vitis9KSNP), which was proposed as an efficient way to differentiate vine species and varieties that they could form the basis for further use in Genome Wide Association (GWA) studies. Three years later, another SNP array was developed (GrapeReSeq 18K Vitis genotyping chip) that included 15.022 SNPs from *Vitis vinifera*, 1.000 SNPs from *Vitis aestivalis*, 1.000 SNPs from *Vitis berlandierii*, 1.000 SNPs from *Vitis labrusca*, 1.000 SNPs from *Vitis cinerea*, 400 SNPs from *Vitis lincecumï*, and 578 SNPs from *Vitis rotundifolia* (Le Paslier et al., 2013). The latter array was utilized by De Lorenzis et al. (2015a) to highlight polymorphisms on Georgian vine samples and by Laucou et al. (2018) who used a large amount of genotypic data to refine the genetic diversity of grapes. Even further, Bianchi et al. (2020) utilized the GrapeReSeq 18K Vitis chip to genotype non-*V. vinifera* populations successfully.

Microarrays are characterized by “ascertainment bias” which result in reduced “flexibility” and “transferability”; markers useful for one family are not always transferable to related families (Hyma et al., 2015). The utilization of NGS offers an alternate approach to SNP microarrays for high-throughput genotyping (Yang et al., 2016). The advent of the former technology bearing the ability to find thousands of SNP per assay has subsequently increased the ability to analyze genomes in cost-effective manner (Marrano et al., 2017).

One of the limitations that the microarrays present is that a prior knowledge of the genome sequence is needed to detect and validate polymorphisms (Marrano et al., 2017), while the cost remains high (Yang et al., 2016) especially if the process is outsourced (Thomson, 2014). On the other hand, NGS strategies don’t need any prior knowledge of the genome as they utilize *de-novo* sequencing (Gambino et al., 2017; Roach et al., 2018; Girollet et al., 2019). Furthermore, no identification and validation of polymorphisms is required in a panel, as it is possible to compare all SNP markers either with respect to a draft reference genome or a re-sequenced reference genome (Liang et al., 2019). Developing new trait-specific SNP for known genes is time consuming, costly, depending on the type of crop and the genetic architecture of the traits (Thomson, 2014).

### 7.2 Next generation sequencing-based strategies

New generation of sequencing (NGS) technologies significantly changed phylogenetic analyses through the increase of the generated data focusing either in genomic level or at specific loci. In any case, the vast array of generated data permits the increase of the number of taxa that can be included in the analyses (Lemmon and Lemmon, 2013). On the other hand, the advantage of higher efficiency and lowered cost per data unit is coupled with the demand for increased computer power for analyses and special bioinformatics skills. Reduced-representation library sequencing (Altshuler et al., 2000) and more specifically, single or double digest restriction-site-associated DNA sequencing (Baird et al., 2008; Peterson et al., 2012) are among the most common work flows.

Such efficient NGS platforms can be used to detect SNP to identify genetic differences not only between varieties but also between clones. For example, Gambino et al. (2017), discriminated *V. vinifera* cv. “Nebbiolo” clones after revealing unique SNP markers while Roach et al. (2018), detected a set of 1620 markers (SNP and indels) that helped them to find differences between 15 clones of the “Zinfadel” variety.

In most of the cases, NGS needs the recruitment of a reference genome to highlight differences and relationships between individuals. Roach et al. (2018) aimed to investigated the genetic diversity in Chardonnay clones after created a high-quality reference genome of the clone I10V1 utilizing the single molecule real-time (SMRT) technology. They showed that Chardonnay contains genomic regions with extensive similarity with Pinot Noir and Gouais blanc leading to the conclusion that they are first degree relatives. In another work, Liang et al. (2019), utilized paired-end re-sequencing and reported the whole-genome analysis of 472 *Vitis* accessions from a wide geographic distribution that helped to identify certain relations between the domesticated grapevines.

The availability of a reference genome is of extreme importance when NGS is in the foreground. It may be useful in the conservation of genetic resources (Girollet et al., 2019) or genetic studies, but also may be used for the provision of additional markers including the development of dense SNP arrays (Roach et al., 2018). Moreover, when the target requires a genome assembly of a heterozygous species, the reference genome provides a secure framework to map the generated reads that usually lead to a better assembly (Dominguez Del Angel et al., 2018). The first two grape reference genomes were created by Jaillon et al. (2007), from a highly heterozygous Pinot Noir, and by Velasco et al. (2007), from a highly homozygous clone of Pinot Noir. Though, the former assembly was haploid with low coverage that does not represent the typical complexity of the genome in commercially available vine varieties while the latter, although diploid, remained highly fragmented, setting difficulties in their use (Roach et al., 2018). Since these first genome announcements, more than two hundred of genomes have been published, readily utilized for reference (Magris et al., 2021), including *V. vinifera* common and feral accessions as well as other *Vitis* species. Among them, a *de-novo* assembled reference genome of *V. riparia* has been created, utilizing a third generation long read NGS technology (Girollet et al., 2019). In combination with a second-generation short read technology, Vondras et al. (2019), created a 225x reference genome for the clone “Zin03” (originated from cultivar Zinfandel).

#### 7.2.1 High-Throughput sequencing of simple sequence repeats

SSR analysis, as previously mentioned, has become a well-established technology for *Vitis* phylogenetic analyses. Various SSR database systems have also been established. Most of these systems use a standard set of nine primer pairs that have been proposed by OIV, although many more have been developed over the years by various research teams (**Supplementary Table 1**). SSR alleles are routinely evaluated using a single or multiplexed PCR followed by capillary electrophoresis-based separation. Such electrophoretic-based techniques for variable-length classification offer sufficient time-and cost-effectiveness improvements but are limited in that they have relatively low throughput and do not yield nucleotide sequence information. Nucleotide variation among SSR alleles may be useful for distinguishing alleles, resolving mixed samples, or even to conduct kinship analyses. High-throughput sequencing technology may provide a more robust platform but is not currently routinely used.

High-throughput microsatellite genotyping came to improve genotyping over classic SSR analyses since HTS gives direct access to microsatellite sequences, allowing unambiguous allele identification and enabling automation through bioinformatics. HTS of microsatellites may provide accurate individual identification and result in significant improvement of genotyping process (De Barba et al., 2017). The main drawbacks of SSR relate to low throughput and automation, difficulty of scoring, and lack of transferability between platforms. PCR aberrations such as stutter bands, exaplo degrees of PCR product adenylation, or uneven DNA movement across runs, for instance, impede standardized and automated allele calling that may produce genotyping mistakes (Guichoux et al., 2011). HTS methods offer a solution to circumvent most of these restrictions (Glenn, 2011).

Allele identification by both the nucleotide sequence and the length of the microsatellite increases the accuracy of allele calling because they are not affected by variation in experimental conditions. Thus, allelic variations, including SNPs, indels, and complex repeat structures, may be determined and described. Moreover, bioinformatics permits the complete automation of the genotyping process, and genotype data are platform independent (Guichoux et al., 2011). Recently, a HTS approach has been used to sequence SSR loci as an alternative method to the traditional method of size analysis by Capillary Electrophoresis (CE), a method that suffers from low data transferability between laboratories. HTS SSR may identify sequence variants within or proximal to SSR loci and sequencing of shorter DNA fragments. Nevertheless, it is possible that SSR with compound repetitive motifs cannot be distinguished (Bornman et al., 2012).

An HTS SSR approach was published by Sarhanova et al. (2018) to genotype hundreds of individuals at several, custom-designed SSR loci, employing multiplex PCR and barcoded primers to separate sequence readings from different samplings. Their primary targets were to generate nucleotide sequence data from SSR loci for numerous non-model plant species as well as SNP and indel variations that would lead to the estimation of genetic diversity statistics. In general, the utilization of NGS allows the full reading of the amplified microsatellite loci and their flanking regions (Vartia et al., 2016). This not only allows the estimation of the size of fragments but also gives the advantage of knowing exactly the microsatellite sequence and co-evaluate any mutations that are located within the microsatellite loci.

By utilizing HTS to genotype microsatellite markers, it is feasible to compare amplicon length and full amplicon information allele calling procedures to determine changes in the quantity of genetic information produced (De Barba et al., 2017). Kunej et al. (2020), compared the two SSR approaches and stated that the HTS, although there is space for improvements in terms of speed, accuracy and price, HTS SSR can replace the CE approach in the years to come. Such an HTS SSR approach may be advantageous, particularly in the case of polyploid species, to resolve allele dosage uncertainty, a problem in which it is impossible to determine how many copies of the allele (one to five) are present, for example, in a hexaploidy species (Cui et al., 2022).

Although HTS SSR have limitations, it is one of the most promising and apparent solutions for genotyping applications and can overcome some of the limitations of conventional microsatellite analysis (Curto et al., 2019). It is anticipated that their use will be widespread for Vitis genotyping and identification.

#### 7.2.2 Reduced representation sequencing

The advent of technical advances in genome sequencing allows the efficient analysis of large numbers of loci dispersed across the genome (Genotyping-By-Sequencing, GBS, Scheben et al., 2017) enabling high-throughput analyses in population genetics (Wang et al., 2020). However, plant genomes present challenges to sequence and analyze especially because they are large and, usually, full of repetitive DNA sequences. Reduced representation sequencing approaches have been developed that offer a rapid, comprehensive, and cost-effective alternative method for such complicated genomes (Barbazuk et al., 2005). They refer to subsets of the genomes to be analyzed that have been constructed reproducibly and include a manageable number of loci. For example, the methylation filtration and high C_o_t (HC) selection methods were relative robust and efficient techniques of this category that are based on gene enrichment. Additionally, there are the bisulfite sequencing approaches that may also improve the assessment of epigenetic diversity (Paun et al., 2019).

Restriction site associated sequencing (RADseq, Peterson, 2008) is among the most common reduced-representation genotyping approaches, where NGS is employed to a fraction of genomic data in such a manner to combine simplicity and cost efficiency in library construction and sequencing. The initial method utilizing a single restriction enzyme (Miller et al., 2007) was followed by a modification, where the sequencing library was constructed after a dual selection of restriction enzymes (ddRAD, Peterson et al., 2012). The NGS of such reduced representation libraries have been successfully implemented for genome-wide SNP discovery in flax (Kumar et al., 2012b).

These approaches lower the cost per-sample and the effort required for analysis. Both RADseq and ddRAD protocols have been widely applied to SNP marker development and genotyping on animals, while it is gaining extreme popularity in the plant world. Ongoing optimization of library preparation has resulted in an easy accessed and utilized way for most of angiosperms (Yang et al., 2016). The ddRAD methods has been employed for the construction of genetic linkage maps in cultivated species, *Vitis vinifera* among them (Zhu et al., 2018; Han et al., 2022), and phylogenomics analyses (Magris et al., 2021). Marrano et al. (2017) aimed to find new SNP in the genome of vine *via* a new RADseq protocol using an NGS platform. That protocol proved to be suitable to detect heterozygosity and to discriminate the *V. sativa* and *V. sylvestris* subspecies of *V. vinifera*.

Recently, a different approach was evaluated, the ISSRseq, in which, the reduced representation sequencing library is constructed by pooling multiple single primer PCR based amplicons (ISSR fragments) from multiple samples (Sinn et al., 2022). ISSRseq method represented a simple method to SNP genotyping of any organism and may be exceptionally useful for population genomics studies. Those who lack extensive experience in wet-laboratory techniques or bioinformatics may benefit from the simplicity of ISSRseq in comparison to other reduced representation sequencing approaches.

#### 7.2.3 Amplicon sequencing

Some years earlier, Yang et al. (2016) developed a new strategy to find trait-associated polymorphic SNP markers in grapevine using a low-cost genotyping workflow, called amplicon sequencing (AmpSeq), aiming primarily at finding polymorphic markers related with major phenotypic characteristics. AmpSeq depends on the multiplexing and sequencing of multiple PCR-amplified sequences, thus allowing genotyping of known and unknown polymorphisms (Fresnedo-Ramírez et al., 2017; Fresnedo-Ramírez et al., 2019).

Although SSRs have been successfully adapted in DNA fingerprinting (Yang et al., 2016), they present a degree of inaccuracy generated by the limited number of the SSR loci utilized, the erroneous amplification of the SSR alleles due to the polymerase slippage during the PCR reaction (Li et al., 2017), or the presence of homoplasy. To overcome the drawbacks of SSRs, a hybrid methodology was developed called AmpSeq-SSR. It is an NGS-based SSR genotyping that offers the parallel amplification of several thousands of SSR loci, as reported for rice (Li et al., 2017). According to this, multiple SSR primer pairs amplify the respective microsatellite regions that are subsequently sequenced by NGS. In the research of Fresnedo-Ramírez et al. (2019), 3105 SSR loci were sequenced, a strategy that distinguished varieties and concluded that AmpSeq is, among other, an accurate and low-cost SSR-genotyping method. A similar approach was applied to genotype cucumber cultivars, called Target SSR-seq, but instead of utilizing thousands of SSRs only 16 were needed for the identification of 382 varieties of cucumbers (Yang et al., 2019). In both cases, the targeted amplification of SSR loci requires the prior knowledge of the flanking regions; an “easy” task for model species as the grape, but difficult for non-model plants where bioinformatics should be employed to extract new primer pairs (Lepais et al., 2020).

AmpSeq is a promising method to make it an appropriate genotyping tool for diverse species, including grape, since it offers flexibility, high-throughput, low-cost, accuracy, and semi-automated analysis (Fresnedo-Ramírez et al., 2019). In general, it allows the investigation of hundreds of genetic markers on thousands of environmental and experimental samples at a greatly reduced level of time and resources.

## 8 Conclusions

In this review we presented the recent molecular methods which have been used to characterize the germplasm of the genus *Vitis*. As *Vitis* is being cultivated since ancient times a more precise and detailed roadmap to fingerprint, conserve and protect the diversity pool should be established. Results show that although a wide array of molecular techniques or statistical methods have been employed, there is no an established roadmap. In the face of climatic changes and the increase of food demand across the globe the new technologies should be incorporated with the human intelligence to create a holistic for *Vitis* cultivation, adaptation, and protection. Nonetheless, it’s always important to consider the limitations of any marker system and to use multiple markers and methods to fully characterize genomic diversity in grape populations.

## Author contributions

ET and FV contributed to conception and design of the study. ET and ST wrote the first draft of the manuscript. All authors contributed to the article and approved the submitted version.
