# Supplementary material for: Vitis vinifera genotyping toolbox to highlight diversity and germplasm identification
Source: Front Plant Sci. 2023 Apr 26;14:1139647. doi: 10.3389/fpls.2023.1139647 (PMC10169827; doi:10.3389/fpls.2023.1139647)
Supplement: Supplementary file 1 [file Table_1.docx]

Supplementary Material

***Vitis vinifera* genotyping toolbox to highlight diversity and germplasm identification**

**Stylianos Tympakianakis^1,†^,** **Emmanouil Trantas^1,2,†,*^, Evangelia V. Avramidou^3^,** **Filippos Ververidis^1,2*^**

^1^Laboratory of Biological and Biotechnological Applications, Department of Agriculture, School of Agricultural Sciences, Hellenic Mediterranean University, Heraklion, Greece

^2^Institute of Agri-Food and Life Sciences, Research Center of the Hellenic Mediterranean University, Heraklion, Greece

^3^Institute of Mediterranean Forest Ecosystems, Hellenic Agricultural Organisation “DIMITRA”, Athens, Greece

*** Correspondence:**Emmanouil A. Trantas, [mtrantas@hmu.gr](mailto:mtrantas@hmu.gr)

Filippos Ververidis, [ververidis@hmu.gr](mailto:ververidis@hmu.gr)

† These authors contributed equally to this work and share first authorship

# Supplementary Table 1. Up to date collection of SSR markers used for Vitis genetic analyses. Nine microsatellite markers that are proposed by the OIV for the identification of vine (OIV-VITI_609 2019) have been marked. Column 3 present the Genbank genomic locus in *Vitis vinifera* (1, PN40024) and *V. riparia* (2, cv Riparia Gloire de Montpellier isolate 1030). *V. vinifera* or *V. riparia* chromosome refers to the chromosome where the SSR locus is located. PCR product size refers to the size of amplicon on the *V. vinifera* or *V. riparia* DNA estimated with the NCBI Primer-Blast tool (Ye, et al. 2012). F, forward primer sequence; R, reverse primer sequence; G, genomic, N/A, not available.

| **SSR marker (OIV code)** | **SSR locus primer pair** | **GenBank record** | **SSR locus coordinates** | ***Vitis vinifera***  **Chromosome #** | ***V. riparia* Chromosome** | **SSR Repeat motif** | **Allele Size (bp) in *Vitis* sp.** | **Number of Alleles**  **(No)** | **Observed Heterozygosity**  **[HO]** | **Expected Heterozygosity**  **[HE]** | **Probability of identity**  **[Pi]** | **References** |
| --- | --- | --- | --- | --- | --- | --- | --- | --- | --- | --- | --- | --- |
| **VVS2 (801)** | F: CAGCCCGTAAATGTATCCATC  R: AAATTCAAAATTCTAATTCAACTGG | 1: NC_012017.3  2: NC_048441.1 | 1: F3909894-3909914  R3910030-3910006  2: F15841543-15841523  R 15841399-15841423 | **11** | **11** | (GA)_22_ | **123–161** | **10-13** | **0,723** | **0,815** | **0.093** | (Thomas and Scott 1993, Hvarleva, et al. 2004, Merkouropoulos, et al. 2015, Guo, et al. 2016, Stavrakaki, et al. 2020) |
| **VVMD5 (802)** | F: CTAGAGCTACGCCAATCCAA  R: TATACCAAAAATCATATTCCTAAA | 1: NC_012022.3  2: NC_048446.1 | 1: F 20785556-20785575  R 20785782-20785759  2: F 22785066-22785085  R 22785330-22785307 | **16** | **16** | (CT)_3_, AT(CT)_11_, ATAG(AT)_3_ | **226-246** | **8** | **0,745** | **0,845** | **0.075** | (Bowers, et al. 1996, Schuck, et al. 2009, Carimi, et al. 2010, Karatas, et al. 2014, Merkouropoulos, et al. 2015, Guo, et al. 2016) |
| **VVMD7 (803)** | F: AGAGTTGCGGAGAACAGGAT  R: CGAACCTTCACACGCTTGAT | 1: NC_012013.3  2: NC_048447.1 | 1: F 1178552-1178571  R 1178797-1178778  2: F 17261888-17261869  R 17261625-17261644 | **7** | **17** | (CT)_14.5_ | **233-263** | **14** | **0,806** | **0,819** | **0.069** | (Bowers, et al. 1996, Schuck, et al. 2009, Merkouropoulos, et al. 2015, Guo, et al. 2016) |
| **VVMD27 (804)** | F: GTACCAGATCTGAATACATCCGTAAGT  R: ACGGGTATAGAGCAAACGGTGT | 1: NC_012011.3  2: NC_048435.1 | 1: F 4472201-4472175  R 4472022-4472043  2: F 4125679-4125653  R 4125474-4125495 | **5** | **5** | (CT)n | **173-194** | **11** | **0,840** | **0,812** | **0.072** | (Bowers, et al. 1999, Schuck, et al. 2009, Merkouropoulos, et al. 2015, Guo, et al. 2016, Marques da Silva, Figueiredo et al. 2020) |
| **VrZAG62 (805)** | F: GGTGAAATGGGCACCGAACACACGC  R: CCATGTCTCTCCTCAGCTTCTCAGC | 1:NC_012013.3  2:NC_048437.1 | N/A | **7** | **7** | (GA)_19_ | **180-207** | **8-10** | **0.8514** | **0,839** | **0.124** | (Sefc, et al. 1999, Hvarleva, et al. 2004, Galbács, et al. 2009, Carimi, et al. 2010, Karatas, et al. 2014, Merkouropoulos, et al. 2015, Guo, et al. 2016, Rustioni, et al. 2016) |
| **VrZAG79 (806)** | F: AGATTGTGGAGGAGGGAACAAACCG  R: TGCCCCCATTTTCAAACTCCCTTCC | 1: NC_012011.3  2: NC_048435.1 | 1: F5692717-5692693  R 5692460-5692484  2: F 5338631-5338607  R 5338374-5338398 | **5** | **5** | (GA)_19_ | **240-264** | **10** | **0.7027** | **0,818** | **0.065** | (Sefc, et al. 1999, Hvarleva, et al. 2004, Halasz, et al. 2005, Galbács, et al. 2009, Carimi, et al. 2010, Merkouropoulos, et al. 2015, Guo, et al. 2016, Rustioni, et al. 2016) |
| **VVMD32 (807)** | F: TATGATTTTTTAGGGGGGTGAGG  R: GGAAAGATGGGATGACTCGC | 1: NC_012010.3  2: NC_048434.1 | 1: F 18035848-18035826  R 18035578-18035597  2: F 18639366-18639344  R 18639131-18639150 | **4** | **4** | (CT)n | **239-273** | **15** | **0,734** | **0,818** | **0.073** | (Bowers, et al. 1999, Schuck, et al. 2009, Laucou, et al. 2011, Merkouropoulos, et al. 2015, Guo, et al. 2016, Marques da Silva, et al. 2020) |
| **VVMD25 (808)** | F: TTCCGTTAAAGCAAAAGAAAAAGG  R: TTGGATTTGAAATTTATTGAGGGG | 1: NC_012017.3  2: NC_048441.1 | 1: F 2971933-2971910  R 2971693-2971716  2: F 16798603-16798626  R 16798843-16798820 | **11** | **11** | (CT)n | **241-259** | **10** | **0,883** | **0,784** | **0.107** | (Galbács, et al. 2009, Schuck, et al. 2009, Laucou, et al. 2011, Merkouropoulos, et al. 2015, Guo, et al. 2016, Marques da Silva, et al. 2020) |
| **VVMD28 (809)** | F: ACAATTCAATGAAAAGAGAGAGAGAGA  R: TCATCAATTTCGTATCTCTATTTGCTG | 1: NC_012009.3  2: NC_048433.1 | 1: F 11617504-11617531  R 11617740-11617714  2: F 11553155-11553128  R 11552939-11552965 | **3** | **3** | (CT)n | **218-278** | **15** | **0,860** | **0,869** | **0.044** | (Galbács, et al. 2009, Schuck, et al. 2009, Laucou, et al. 2011, Merkouropoulos, et al. 2015, Guo, et al. 2016, Marques da Silva, et al. 2020) |
| **ccSSR-14 a** | F: GGGTATAATGGTAGATGCCC  R: GCCGTAGTAAATAGGAGAGAAA | 1:NC_007957.1  2:NC_039680.1 | 1: F 89061- 89080  R 89262- 89241  2: F 89088- 89107  R 89290- 89269 | **N/A** | **N/A** | (T)_14_ | **201-204** | **N/A** | **N/A** | **N/A** | **N/A** | (Sefc, et al. 2009) |
| **Scu04vv** | F: TGTCCTCTTTCCCTCTCCCAAC  R: CAGTCTGTCATCTGACCATGTAGCC | 1:NC_012007.3  2:NC_048431.1 | 1: F 4261254-4261275  R 4261428-4261404  2: F 4354617-4354638  R 4354789-4354765 | **1** | **1** | (CT)_8_ | **167-177** | **3** | **0.896** | **0.574** | **0.459** | (Scott, et al. 2000, Ekhvaia, et al. 2014) |
| **Scu05vv** | F: CAAGCAGTTATTGAAGCTGCAAGG  R: TCATCCATCACACAGGAAACAGTG | 2:NC_048442.1 | 2: F 15237440-15237417  R 15237277-15237301 | **N/A** | **12** | (AT)_13_ | **134-214** | **15** | **0.30** | **0.67** | **N/A** | (Scott, et al. 2000, Drábek, et al. 2016, Italian_Vitis_Database 2022) |
| **Scu06vv** | F: CCCTAGTCTCTCTACCTATCCATG  R: CCTAATGCCAGGAAGGTTGC | 1:NC_012023.3  2:NC_048447.1 | 1: F 3158905-3158882  R 3158735-3158754  2: F 17721673-17721696  R 17721876-17721857 | **17** | **17** | (AT)_8_ | **131-211** | **2** | **0.250** | **N/A** | **0.212** | (Scott, et al. 2000, Jahnke, et al. 2011, Italian_Vitis_Database 2022) |
| **Scu07vv** | F: CCGAAGAGGAATATGGGTTTGAG  R: CCTAACTTGAAACGAAAGGACTGC | 1:NC_012021.3  2:NC_048445.1 | 1: F 16971038-16971016  R 16970836-16970859  2: F 3760003-3760025  R 3760208-3760185 | **15** | **15** | (ACC)_5_ | **163-243** | **4** | **N/A** | **N/A** | **N/A** | (Scott, et al. 2000, Wang, et al. 2015, Italian_Vitis_Database 2022) |
| **Scu08vv** | F: CGAGACCCAGCATCGTTTCAAG  R: GCAAAATCCCCGTACAAGTC | 1:NC_012017.3  2:NC_048441.1 | 1: F 2579555-2579534  R 2579373-2579392  2: F 17193768-17193789  R 17193944-17193925 | **11** | **11** | (GGT)_5_ | **185-197** | **4** | **0.182** | **0.197** | **0.685** | (Scott, et al. 2000, Halasz, et al. 2005, Galbács, et al. 2009) |
| **Scu09vv** | F: AAGCAGCAGTTATTGGCG  R: CAGATACTGAGGGTTTAAGCTC | 1: NC_012020.3  2: NC_048444.1 | 1: F 24477739-24477722  R 24474623-24474644  2: F 5860287-5860304  R 5863386-5863365 | **14** | **14** | (GGT)_5_ | **82-162** | **N/A** | **N/A** | **N/A** | **N/A** | (Scott, et al. 2000, Italian_Vitis_Database 2022) |
| **Scu10vv** | F: TACCCCCACAACCCTTTT  R: TTCTCCGCCACCTCCTTTTCAC | 1:NC_012024.3  2:NC_048448.1 | 1: F 4520661- 4520678  R 4520877- 4520856  2: F 4606282- 4606299  R 4606489- 4606468 | **18** | **18** | (CAA)_6_ | **202-217** | **6** | **0.182** | **0.722** | **0.124** | (Scott, et al. 2000, Halasz, et al. 2005, Galbács, et al. 2009) |
| **Scu11vv** | F: AATTGATAGTGCCACGTTCTCGCC  R: AACGCCGACAAGAATCCCAAGG | 1:NC_012025.3  2:NC_048449.1 | 1: F 6614205- 7003606  R 7003391- 7003412  2: F 6614205- 6614182  R 6613961- 6613982 | **19** | **19** | (CTT)_8_ | **213-293** | **25** | **0.874** | **N/A** | **0.630** | (Scott, et al. 2000), |
| **Scu14vv** | F: CTGCACTTGAATACGAGCAGGTC  R: TGTTATATGATCCTCCCCCTCCTC | 1:NC_012022.3  2:NC_048446.1 | N/A | **16** | **16** | (GAA)_6_ | **168-188** | **4** | **N/A** | **N/A** | **N/A** | (Scott, et al. 2000, Dauob, et al. 2018) |
| **Scu15vv** | F: GCCTATGTGCCAGACCAAAAAC  R: TTGGAAGTAGCCAGCCCAACCTTC | 1:NC_012020.3  2: NC_048444.1 | N/A | **14** | **14** | (GAA)_6_ | **155-235** | **N/A** | **N/A** | **N/A** | **N/A** | (Scott, et al. 2000, Italian_Vitis_Database 2022) |
| **Scu16vv** | F: CAAAGACAAAGAAGCCACCGAC  R: ACCCTCTAAAGCACACACAGGAAC | 1:NC_012024.3  2:NC_048448.1 | N/A | **18** | **18** | (GAA)_5_ | **130-210** | **N/A** | **N/A** | **N/A** | **N/A** | (Scott, et al. 2000, Italian_Vitis_Database 2022) |
| **UCH11** | F: ATGCCCGAGAAGAGTCGAGAA  R: CTGCCGTTTGGGTAAGATGCT | 1:NC_012011.3  2: NC_048435.1 | N/A | **5** | **5** | (GA)_15_ | **220–262** | **21** | **0.7977** | **0.7968** | **0.191** | (Lefort and Roubelakis-Aggelakis 2000, Lefort, et al. 2002, Hvarleva, et al. 2004) |
| **UCH12** | F: TTTTCATTGAAAAGAAGGA  R: TGTGCTTTGTGCTAGATAA | 1:NC_012020.3  2:NC_048444.1 | N/A | **14** | **14** | (CT)_17_(CA)_13_ | **135–234** | **21** | **0.9315** | **0.9047** | **N/A** | (Lefort, et al. 2002) |
| **UCH19** | F: GATTTGAAAGTCGAAAGCCAGG  R: TGCAAAGACTGTGAGATGAGGG | 1:NC_012007.3  2:NC_048431.1 | N/A | **1** | **1** | (CT)_23_ | **174–212** | **12** | **0.8630** | **0.8646** | **N/A** | (Lefort, et al. 2002) |
| **UCH2** | F: AGCTCGGCTAGCTGCAAAATC  R: ACCCTTCCCTCTTCAAAACCC | 1:NC_012025.3  2:NC_048449.1 | N/A | **19** | **19** | (AG)_15_ | **146–200** | **19** | **0.7638** | **0.9023** | **N/A** | (Lefort, et al. 2002), |
| **UCH29** | F: AAACATGATCTGATGCAGGTGA  R: CAACCTGTTGATGAAAGGGAAA | 1:NC_012007.3  2:[NC_048431.1](https://www.ncbi.nlm.nih.gov/nucleotide/1847502376?from=21451094&to=21451382&report=gbwithparts) | N/A | **1** | **1** | (CT)_18_ | **207–315** | **26** | **0.7932** | **0.8458** | **0.116** | (Lefort, et al. 2002, Hvarleva, et al. 2004) |
| **UCH35** | F: AAATGTGCAAGTTGAAGAGGGA  R: AGACCGTTCAAACAAGCAAATG | N/A | N/A | **N/A** | **N/A** | (CT)_17_ | **133–181** | **15** | **0.7313** | **0.8567** | **N/A** | (Lefort, et al. 2002) |
| **UCH40** | F: GCAGTTGATGCAAAACAACAGT  R: CACATCATTCATTGATGAGGCT | 1:NC_012019.3  2:NC_048443.1 | N/A | **13** | **13** | Imperfect run of  (GCA)n(ACA)m | **237–312** | **17** | **0.8636** | **0.8846** | **N/A** | (Lefort, et al. 2002) |
| **VChr10a** | F: AAATGTTTAGTAGCCTCATTTTGTTT  R: TTTGTTCGGAACTACTCTTCTTCA | 1:NC_012016.3  2:NC_048440.1 | N/A | **10** | **10** | ACT | **98-137** | **8** | **0.271** | **0.647** | **0.618** | (Cipriani, et al. 2008, Italian_Vitis_Database 2022) |
| **VChr10b** | F: CCATGTCCAACCGAAACAAC  R: CAGAAATCTCGTGTCGCTCA | 1:NC_012016.3  2:NC_048440.1 | N/A | **10** | **10** | AAC | **116-136** | **5** | **0.813** | **0.703** | **0.635** | (Cipriani, et al. 2008, Italian_Vitis_Database 2022) |
| **VChr11a** | F: GGGATAAGGTGAAAGCCTCA  R: ATGCTTGGTATCTGGCAACC | 1:NC_012017.3  2:NC_048441.1 | N/A | **11** | **11** | AAAG | **178-207** | **6** | **0.542** | **0.558** | **0.506** | (Cipriani, et al. 2008, Italian_Vitis_Database 2022) |
| **VChr11b** | F: TGAGTTGAGCTATTGGCTTTGA  R: AGCAACTCTGTCCATCCATGT | 1:NC_012017.3 | N/A | **11** | **N/A** | AGAT | **151-163** | **5** | **0.690** | **0.770** | **0.720** | (Cipriani, et al. 2008, Italian_Vitis_Database 2022) |
| **VChr12a** | F: GCTTTAAATGTTAGATTAGGGCACTC  R: TCCATGTTGTTTGCTCTTTCC | 1:NC_012018.3  2:NC_048442.1 | N/A | **12** | **12** | AATT | **126-146** | **7** | **0.542** | **0.695** | **0.640** | (Cipriani, et al. 2008, Italian_Vitis_Database 2022) |
| **VChr12b** | F: AAACACAAGGTTGCATTGGA  R: GGCTTTCTTGTGGACTTAAATGA | 1:NC_012018.3  1:NC_012014.3  2:NC_048438.1 | N/A | **12, 8** | **8** | AATT | **161-169** | **2** | **0.250** | **0.449** | **0.346** | (Cipriani, et al. 2008, Italian_Vitis_Database 2022) |
| **VChr13a** | F: TGGCAGAGCAAATGAATCAA  R: TTGGATGGATTGGAATGACC | 1:NC_012019.3  2:NC_048443.1 | N/A | **13** | **13** | AAAAG | **135-165** | **7** | **0.625** | **0.698** | **0.652** | (Cipriani, et al. 2008, Italian_Vitis_Database 2022) |
| **VChr13b** | F: TAAGCATTCTGGGCTTTTCC  R: TCGTCTATATGCGACCTTGG | 1:NW_003724181.1  2:NC_048443.1 | N/A | **13** | **13** | AAAT | **145-170** | **8** | **0.500** | **0.643** | **0.613** | (Cipriani, et al. 2008, Italian_Vitis_Database 2022) |
| **VChr13c** | F: AGACCCAAGGGCAAGGTACT  R: AACACCGTTAGGCATACTCCA | 1:NC_012019.3  2:NC_048443.1 | N/A | **13** | **13** | AAT | **114-135** | **5** | **0.750** | **0.744** | **0.689** | (Cipriani, et al. 2008, Italian_Vitis_Database 2022) |
| **VChr13d** | F: AATCTGACGCCATGAGGAAG  R: TCGTCTATATGCGACCTTGG | N/A | N/A | **N/A** | **N/A** | AATC | **174-191** | **4** | **0.250** | **0.248** | **0.234** | (Cipriani, et al. 2008, Italian_Vitis_Database 2022) |
| **VChr14a** | F: AACCTGGGATGCTGAGAATG  R: TGCATGCATATGGATCTTGT | 1:NC_012020.3  2:NC_048444.1  2:NC_048447.1 | N/A | **14** | **14, 17** | AATC | **128-189** | **3** | **0.500** | **0.541** | **0.444** | (Cipriani, et al. 2008, Italian_Vitis_Database 2022) |
| **VChr14b** | F: CAATTGAACACTTACACTCACAATCA  R: TGTGACTAAAGGTTATTAGCAGGA | 1:NC_012020.3  2:NC_048444.1 | N/A | **14** | **14** | ATC | **176-243** | **15** | **0.234** | **0.811** | **0.786** | (Cipriani, et al. 2008, Italian_Vitis_Database 2022) |
| **VChr15a** | F: CAATCCCAACAGTTCCATGA  R: CGTTTTCTCCTTCGGACAAG | 1:NC_012021.3 | N/A | **15** | **N/A** | ATCC | **127-165** | **8** | **0.717** | **0.728** | **0.677** | (Cipriani, et al. 2008, Italian_Vitis_Database 2022) |
| **VChr15b** | F: GGGTCCAATTCCTTTTGGTT  R: CGAAAGACTCAATTGCCACA | 1:NC_012021.3  2:NC_048445.1 | N/A | **15** | **15** | AAT | **90-151** | **10** | **0.292** | **0.830** | **0.799** | (Cipriani, et al. 2008, Italian_Vitis_Database 2022) |
| **VChr16a** | F: TTCATGTGTGACACCCCTTT  R: AATGTCCATGCTTCAAAATACC | 1:NC_012022.3 | N/A | **16** | **N/A** | AAAT | **100-167** | **8** | **0.604** | **0.632** | **0.602** | (Cipriani, et al. 2008, Italian_Vitis_Database 2022) |
| **VChr16b** | F: ATAAGGCGCTGACTTTGTGA  R: CCAGGAGATCAACCACCATT | 1:NC_012022.3 | N/A | **16** | **N/A** | AATT | **165-193** | **7** | **0.500** | **0.558** | **0.526** | (Cipriani, et al. 2008, Italian_Vitis_Database 2022) |
| **VChr16c** | F: TTTCAATATTCCAAATGTGACCT  R: CATTTCTTTGCTCTTCCTGCT | 1:NC_012022.3  2:NC_048446.1 | N/A | **16** | **16** | AATT | **151-161** | **4** | **0.638** | **0.582** | **0.516** | (Cipriani, et al. 2008, Italian_Vitis_Database 2022) |
| **VChr17a** | F: AGGAAGAGGATTGATCACCA  R: GTGCCAACCCTTGCACTATT | 1:NC_012023.3 | N/A | **17** | **N/A** | AACC | **170-184** | **3** | **0.178** | **0.463** | **0.372** | (Cipriani, et al. 2008, Italian_Vitis_Database 2022) |
| **VChr17b** | F: CCAAAGCCGACAACTTCTTC  R: CCGCCATAAACCCTAAACCT | 1:NC_012023.3 | N/A | **17** | **N/A** | ACTC | **154-162** | **3** | **0.021** | **0.142** | **0.134** | (Cipriani, et al. 2008, Italian_Vitis_Database 2022) |
| **VChr17c** | F: CCATGTTCCATCCCACTTCT  R: CGTACGTACAAAATCTTGGGATAC | 1:NC_012023.3 | N/A | **17** | **N/A** | AAT | **94-120** | **8** | **0.333** | **0.548** | **0.519** | (Cipriani, et al. 2008, Italian_Vitis_Database 2022) |
| **VChr18a** | F: TTCCCACCCGGTAAATATGA  R: CATCCAAACATCACGCTGAG | 1:NC_012024.3  2:NC_048448.1 | N/A | **18** | **18** | AAGG | **151-191** | **8** | **0.542** | **0.705** | **0.659** | (Cipriani, et al. 2008, Italian_Vitis_Database 2022) |
| **VChr18b** | F: ATACGCAAATGATCACAGCA  R: CATTTTCTCCATGGCCTCAT | 1:NC_012024.3  2:NC_048448.1 | N/A | **18** | **18** | AGGC | **137-154** | **5** | **0.521** | **0.730** | **0.672** | (Cipriani, et al. 2008, Italian_Vitis_Database 2022) |
| **VChr18c** | F: TGAAGCCCATTACAACCAAA  R: TGCAAATTAAAGCCAAGTGTG | 1:NC_012024.3 | N/A | **18** | **N/A** | AATC | **125-134** | **4** | **0.208** | **0.196** | **0.187** | (Cipriani, et al. 2008, Italian_Vitis_Database 2022) |
| **VChr18d** | F: TAGGTACGGTCCCAATGACC  R: TCGATCGATCATCTTCATCTCT | 1:NW_003724189.1 | N/A | **18** | **N/A** | AAACT | **195-205** | **3** | **0.364** | **0.411** | **0.370** | (Cipriani, et al. 2008, Italian_Vitis_Database 2022) |
| **VChr19a** | F: TTTGTTAGGTGTTGTTACCCGTTA  R: ATCTTCTGGCCATGTGGTTC | 1:NC_012025.3 | N/A | **19** | **N/A** | AAG | **121-150** | **10** | **0.792** | **0.784** | **0.748** | (Cipriani, et al. 2008, Italian_Vitis_Database 2022) |
| **VChr19b** | F: TGGATTCACCATTGTCCTCA  R: CGAGGATACCAACAAGAATGAA | 1:NC_012025.3  2:NC_048449.1 | N/A | **19** | **19** | AGAT | **157-171** | **5** | **0.500** | **0.711** | **0.652** | (Cipriani, et al. 2008, Italian_Vitis_Database 2022) |
| **VChr1a** | F: TTCATACCTTGCAGGGAGCTA  R: TGATTTCCATTCCCAAATTCA | 1:NC_012007.3 | N/A | **1** | **N/A** | ATCC | **175-244** | **9** | **0.458** | **0.545** | **0.525** | (Cipriani, et al. 2008, Italian_Vitis_Database 2022) |
| **VChr1b** | F: AGATGGGTGGCATTAGCAAG  R: TTATTTCCCTCCCTCGCTGT | 1:NC_012007.3  2:NC_048431.1 | N/A | **1** | **1** | ATCC | **90-111** | **6** | **0.771** | **0.689** | **0.635** | (Cipriani, et al. 2008, Italian_Vitis_Database 2022) |
| **VChr1c** | F: CTGGCCTTATGCACAAAGTG  R: GATGAACACATCAATCAAATACCC | 1:NC_012007.3  2:NC_048431.1 | N/A | **1** | **1** | AGCC | **87-100** | **3** | **0.521** | **0.476** | **0.369** | (Cipriani, et al. 2008, Italian_Vitis_Database 2022) |
| **VChr2a** | F: GGTCCGCTTTTGAGAAGAAA  R: CATGTGAACGCGCTAAACAC | 1:NC_012008.3  2:NC_048432.1 | N/A | **2** | **2** | AGGC | **137-155** | **3** | **0.542** | **0.405** | **0.328** | (Cipriani, et al. 2008, Italian_Vitis_Database 2022) |
| **VChr2b** | F: CCTCCTGCGAACAAGTCTGT  R: GTTGCTGGATTTGTGGAAGG | 1:NC_012008.3  2:NC_048432.1 | N/A | **2** | **2** | AGCT | **112-128** | **6** | **0.563** | **0.508** | **0.467** | (Cipriani, et al. 2008, Italian_Vitis_Database 2022) |
| **VChr2c** | F: CTCAAAGCCCTCCAATTCAA  R: GGGCTCATGTGTCTGGAGTT | 1:NC_012008.3  2:NC_048432.1 | N/A | **2** | **2** | AGCC | **147-158** | **5** | **0.521** | **0.490** | **0.443** | (Cipriani, et al. 2008, Italian_Vitis_Database 2022) |
| **VChr3a** | F: CAATCATATGAGCAAGGCATGT  R: GCTTCCTGAAATTTGTGTCCA | 1:NC_012009.3  2:NC_048433.1 | N/A | **3** | **3** | AAT | **175-249** | **14** | **0.688** | **0.836** | **0.810** | (Cipriani, et al. 2008, Italian_Vitis_Database 2022) |
| **VChr4a** | F: CAACTGGGATCCAAGACCTC  R: CAGCTTCACAGGTAACCACA | 1:NC_012010.3  2:NC_048434.1 | N/A | **4** | **4** | AAAG | **173-203** | **7** | **0.563** | **0.644** | **0.585** | (Cipriani, et al. 2008, Italian_Vitis_Database 2022) |
| **VChr5a** | F: ACTTGGCGAGTATTTGTTCTAAA  R: CCGCTTTGTGAAGGTATCCA | 1:NC_012011.3  2:NC_048435.1 | N/A | **5** | **5** | AGATG | **183-259** | **11** | **0.750** | **0.772** | **0.745** | (Cipriani, et al. 2008, Italian_Vitis_Database 2022) |
| **VChr5b** | F: CTTCTCGGTCATGGTCATTG  R:CTCCTTCCACCTCTGGTTCA | 1:NC_012011.3  2:NC_048435.1 | N/A | **5** | **5** | AAAG | **179-219** | **10** | **0.750** | **0.792** | **0.754** | (Cipriani, et al. 2008, Italian_Vitis_Database 2022) |
| **VChr5c** | F: CCCATCAGTTTGCCTATGAA  R: TTTGATCTTGTTATTGTGCTGTTAC | 1:NC_012011.3  2:NC_048435.1 | N/A | **5** | **5** | ACAT | **83-123** | **7** | **0.729** | **0.747** | **0.704** | (Cipriani, et al. 2008, Italian_Vitis_Database 2022) |
| **VChr6a** | F: AATGTTGAGCTTTGGGCTTG  R: CCAATTCTTCCATACCTCAAAA | 1:NC_012012.3 | N/A | **6** | **N/A** | AATC | **173-180** | **4** | **0.532** | **0.572** | **0.502** | (Cipriani, et al. 2008, Italian_Vitis_Database 2022) |
| **VChr7a** | F: TCCGTGTCACAAAGAACATGA  R: ATTAGGGCACTGCCTCTTCC | 1:NC_012013.3 | N/A | **7** | **N/A** | AAAAG | **126-140** | **3** | **0.417** | **0.506** | **0.386** | (Cipriani, et al. 2008, Italian_Vitis_Database 2022) |
| **VChr7b** | F: AAAGGGCCTAAACTCTTAATAACTTG  R: TGCTTTATAGACACTAACCCACAAA | 1:NW_003724159.1  2:NC_048437.1 | N/A | **7** | **7** | ACAT | **172-195** | **6** | **0.688** | **0.703** | **0.651** | (Cipriani, et al. 2008, Italian_Vitis_Database 2022) |
| **VChr7c** | F: CACTTCTCTGCCACCCATTT  R: GGTTGGAAATTCTAGGGCATT | 1:NC_012013.3 | N/A | **7** | **N/A** | ATGC | **101-108** | **3** | **0.688** | **0.620** | **0.532** | (Cipriani, et al. 2008, Italian_Vitis_Database 2022) |
| **VChr8a** | F: ACCCACTGCCACTCTCTCAT  R: AAATCTCCGGGATCCTTTTG | 1:NC_012014.3  2:NC_048438.1 | N/A | **8** | **8** | AAT | **172-206** | **12** | **0.596** | **0.835** | **0.805** | (Cipriani, et al. 2008, Italian_Vitis_Database 2022) |
| **VChr8b** | F: TGTGTGATGTTTTGTCGATGG  R: TGAACCAAGTTCTAATTTACATTTCC | 1:NC_012014.3 | N/A | **8** | **N/A** | AAG | **58-156** | **16** | **0.646** | **0.889** | **0.870** | (Cipriani, et al. 2008, Italian_Vitis_Database 2022) |
| **VChr9a** | F: GCGACAGCATCACTTCAATC  R: GAATTGCCAAGGACAAGGAG | 1:NC_012015.3  2:NC_048439.1 | N/A | **9** | **9** | AAG | **87-117** | **8** | **0.787** | **0.809** | **0.776** | (Cipriani, et al. 2008, Italian_Vitis_Database 2022) |
| **VChr9b** | F: AGCGTCATGACAGGTATCAGAA  R: AAAGAATTAATCATTACCATTTCACG | 1:NC_012015.3 | N/A | **9** | **N/A** | AAT | **102-160** | **10** | **0.313** | **0.865** | **0.840** | (Cipriani, et al. 2008, Italian_Vitis_Database 2022) |
| **VMC1b11** | F: CTTTGAAAATTCCTTCCGGGTT  R: TATTCAAAGCCACCCGTTCTCT | N/A | N/A | **N/A** | **N/A** | (AG)_17_ | **167-196** | **8** | **0.947** | **0.809** | **0.771** | (Cipriani, et al. 2008, Laucou, et al. 2011, Dos Anjos 2013; Karatas, et al. 2014, Zarouri 2016, Nebish, et al. 2017) |
| **VMC1C10** | F: ATATTCACAGCTGTTCCAAGTCCCA  R: GAGAGTGGCGGAAGGCTTGTTGACC | N/A | N/A | **N/A** | **N/A** | (TGC)_6_ | **129-185** | **16** | **0.841** | **0.863** | **0.033** | (Jahnke, et al. 2011, Dos Anjos 2013; Zarouri 2016) |
| **VMC1E8** | F: CAGCGAGCTCTTGATTTATTGT R: GATCATAGCTTCAACGGCTTTT | N/A | N/A | **N/A** | **N/A** | (GA)_21_ | **206-231** | **10** | **0.773** | **0.805** | **0.063** | (Dos Anjos 2013; Guo, et al. 2016, Zarouri 2016) |
| **VMC1F10** | F: CATACAAGGAATTTACCCCCA  R: ACCTCTTGTGCTGTCTAACCA | N/A | N/A | **N/A** | **N/A** | (AG)_18_ | **190-208** | **10** | **0.643** | **0.816** | **0.056** | (Dos Anjos 2013; Zarouri 2016) |
| **VMC2H4** | F: AGTACCAGGTGTGCCTATAAGAATC  R: GTTGATTGGATGTTCCAGAGAGGAT | N/A | N/A | **N/A** | **N/A** | N/A | **118-237** | **15** | **0.816** | **0.86** | **0.033** | (Dos Anjos 2013; Zarouri 2016) |
| **VMC3B12** | F: ATAAGGCAGGTTGATTACAGGA  R: CATCACAGGTTGATTCGACACT | N/A | N/A | **N/A** | **N/A** | (AG)_24_ | **N/A** | **N/A** | **N/A** | **N/A** | **N/A** | (Dos Anjos 2013; Guo, et al. 2016) |
| **VMC3C9** | F: ATAAAATGGAATTAAGGGGGGA R: CAAACGCTAGATACCATGGAGA | N/A | N/A | **N/A** | **N/A** | N/A | **N/A** | **N/A** | **N/A** | **N/A** | **N/A** | (Gaspero, et al. 2000, Guo, et al. 2016) |
| **VMC3D12** | F: TGTCACTGTGGACATAGGGAG  R: ATCACCAAAGGGAAGCAAAAG | 1: NC_012019.3  2: NC_048443.1 | 1:F 083896- 8083876  R 8083693- 8083713  2:F 20214024- 20214044  R 20214226- 20214206 | **13** | **13** | (TC)_21_ | **197-251** | **23** | **0.841** | **0.871** | **0.856** | (Dos Anjos 2013; Zarouri 2016) |
| **VMC4A1** | 5′ATGCGACCTTAATAAATTGGGAA  5′AAGCTAGGCTTGTATGAGGGAGA | N/A | N/A | **N/A** | **N/A** | (AG)_20_ | **N/A** | **N/A** | **N/A** | **N/A** | **N/A** | (Gaspero, et al. 2000) |
| **VMC4A5** | 5′ATTTTCCACAGGCAAACCACAT  5′TGTGGTTGTTGTAGCCTATCGG | 2:NC_048439.1 | N/A | **N/A** | **9** | (AGAC)_5_N_31_(AG)_14_ | **N/A** | **N/A** | **N/A** | **N/A** | **N/A** | (Gaspero, et al. 2000) |
| **VMC4C6** | 5′CTCCATCCCTATCTCATCAG  5′CTCTAACACCCAATCTCACA | 1:NC_012011.3 | N/A | **5** | **N/A** | (GCT)_11_ | **153-177** | **7** | **0.696** | **0.676** | **0.153** | (Gaspero, et al. 2000, Zarouri 2016) |
| **VMC4D2** | 5′TGCAGATACCACATACCCACCT  5′AACAGCAAACATCCCAACTCAG | 1:NC_012007.3  2:NC_048431.1 | N/A | **1** | **1** | (AG)_16_ | **N/A** | **N/A** | **N/A** | **N/A** | **N/A** | (Gaspero, et al. 2000) |
| **VMC4D4** | 5′GTCTTGTAATGGAACCAACTGC  5′AGATTGACCTGGACCTGAAACT | 1:NC_012010.3  2:NC_048434.1 | N/A | **4** | **4** | (GCT)_9_ | **152-176** | **7** | **0.594** | **0.558** | **0.238** | (Gaspero, et al. 2000, Zarouri 2016) |
| **VMC4f3** | F: AAAGCACTATGGTGGGTGTAAA  R: TAACCAATACATGCATCAAGGA | 1:NC_012018.3  2:NC_048442.1 | N/A | **12** | **12** | (AG)_20_AA(AG)_9_ | **165–208** | **12** | **0.868** | **0.874** | **0.848** | (Gaspero, et al. 2000, Laucou, et al. 2011, Nebish, et al. 2017) |
| **VMC4G6** | 5′CCTTGAAGAGATGAGTTTGCTA  5′TATTTAACTTTGTGCCTCTGCT | 1:NC_012012.3  2:NC_048436.1 | N/A | **6** | **6** | (AG)_17_ | **119-139** | **11** | **0.657** | **0.794** | **0.07** | (Gaspero, et al. 2000, Zarouri 2016) |
| **VMC4H5** | 5′GATTTGTGACACTTGTGTAGCG  5′CAAGTGGAAAGCAATCTAGGAA | N/A | N/A | **N/A** | **N/A** | (AG)_5_TG(AG)_33_ | **N/A** | **N/A** | **N/A** | **N/A** | **N/A** | (Gaspero, et al. 2000) |
| **VMC4H6** | 5′GTATAGAACCACGCATCCAACA  5′CCCTTAGTTTCCTCGTGCTTTT | 1:NC_012015.3  2:NC_048439.1 | N/A | **9** | **9** | (AG)_23_ | **N/A** | **N/A** | **N/A** | **N/A** | **N/A** | (Gaspero, et al. 2000) |
| **VMC5E9** | F: ATCCAGAGCCATAACAGATTCA  R: TCACAGCTTTCTCATTACCCTT | 1: NC_012025.3  2: NC_048449.1 | 1:F 4183347- 4183326  R 4183125- 4183146  2:F 4486656- 4486677  R 4486860- 4486839 | **19** | **19** | (AG)_22_(CAC)_6_ | **282-314** | **19** | **0.816** | **0.894** | **0.076** | (Jahnke, et al. 2011; Dos Anjos 2013) |
| **VMC5G8** | F: GCACATGCACATCTTGTTTCACTCT  R: GAGACTTTTGGAAGCAATGATGGCA | N/A | N/A | **N/A** | **N/A** | (CTG)_7_(AG)_11_ | **281-319** | **11** | **0.739** | **0.778** | **0.085** | (Jahnke, et al. 2011, Dos Anjos 2013; Zarouri 2016) |
| **VMC6B11** | F: TGATTATGGCAATAATCACACC  R: TTGCTTACCCATCAAAAAGAAA | 1:NC_012008.3  2:NC_048432.1 | N/A | **2** | **2** | (TC)_20_ | **83-116** | **N/A** | **N/A** | **N/A** | **N/A** | (Arroyo-García and Martínez-Zapater 2004) |
| **VMC6C10** | F: TTCCTGCGAATTCTAACCCCTT  R: CCACTTCCATTCCCTCTCCTGT | 1:NC_012020.3  2:NC_048444.1 | N/A | **14** | **14** | (GA)_17_ | **105-143** | **N/A** | **N/A** | **N/A** | **N/A** | (Arroyo-García and Martínez-Zapater 2004) |
| **VMC6C7** | F: ACATATATCCGAAAGTGTGGGGC  R: CTTAAAGCTTGAAGCTTTTGGTGC | N/A | N/A | **N/A** | **N/A** | (GA)_10_ | **114-161** | **N/A** | **N/A** | **N/A** | **N/A** | (Arroyo-García and Martínez-Zapater 2004) |
| **VMC6D12** | F: CTCTCTTTTCCGAAATTGGGGT  R: ATTTTCCCTGGAAACAAAGTGG | 1:NC_012015.3  2:NC_048439.1 | 1: F 3792236- 3792257  R 3792401- 3792380  2: F 4056320- 4056341  R 4056487- 4056466 | **9** | **9** | (TC)_18_ | **131-181** | **13** | **0.826** | **0.827** | **0.046** | (Arroyo-García and Martínez-Zapater 2004, Zarouri 2016) |
| **VMC6E10** | F: CTAGGTGTGCCAAGAGATCAGA  R: CATTTGTGGGTAGTTGTGAGGA | 1:NC_012011.3  2:NC_048435.1 | 1: F 14299510- 14299531  R 14299606- 14299585  2: F 14323591- 14323612  R 14323699- 14323678 | **5** | **5** | (GA)_13_ | **90-122** | **N/A** | **N/A** | **N/A** | **N/A** | (Arroyo-García and Martínez-Zapater 2004) |
| **VMC6G10** | F: CATCATTCATCCAAATTATGTAG  R: TTTAGTAGGTTAGGGATACCAGT | 1: NC_012010.3 | 1: F 23105311- 23105333  R 23105488-23105466 | **4** | **N/A** | (GA)_14_ | **121-195** | **12** | **0.614** | **0.618** | **0.582** | (Arroyo-García and Martínez-Zapater 2004, Zarouri 2016) |
| **VMC6G8** | F: GAGTGTCAGTCTCAAAATAAGGA  R: CCCCTCATCTCTTCTCTATCTAA | N/A | N/A | **N/A** | **N/A** | (GA)_15_ | **88-109** | **N/A** | **N/A** | **N/A** | **N/A** | (Arroyo-García and Martínez-Zapater 2004) |
| **VMC7G3** | F: ATATTACTAGTGCTGTCCTGCTCCA  R: TGAAAGTTGAAAGAGAGGAAGCAAA | N/A | N/A | **19** | **N/A** | (TC)_16_ | **115-161** | **17** | **0.585** | **0.587** | **0.186** | (Jahnke, et al. 2011, Zarouri 2016) |
| **VMC7h3** | F: TCAGATATTGAAGAACACCACA  R: ACTAGAAAATGCACAATCTCCC | 1: NC_012010.3  2: NC_048434.1 | 1: F 4719044- 4719065  R 4719178- 4719157  2: F 4968212- 4968233  R 4968352- 4968331 | **4** | **4** | (TC)_16_ | **119-175** | **21** | **0.749** | **0.783** | **0.075** | (Dos Anjos 2013; Jing, et al. 2013, Zarouri 2016) |
| **VMC8F10** | F: TATGAAAGATGAATGGCTGCTC  R: AAGGGTGCTTGAAGGTTTATGT | 1: NC_012009.3 | 1: F 3324131- 3324152  R 3324340- 3324319 | **3** | **N/A** | (TC)_19_ | **199-237** | **14** | **0.787** | **0.808** | **0.06** | (Dos Anjos 2013; Guo, et al. 2016, Zarouri 2016) |
| **VMC9a2.1** | F: AGCTCGGCTAGCTGCAAAATC  R: ACCCTTCCCTCTTCAAAACCC | 1: NC_012025.3  2: NC_048449.1 | 1:F 854676- 854656  R 854511- 854531  2:F 765199- 765179  R 765048- 765068 | **19** | **19** | (AG)_15_ | **N/A** | **N/A** | **N/A** | **N/A** | **N/A** | (Dos Anjos 2013; Jing, et al. 2013) |
| **VMCNG1E1** | F: TGTGTTACGCCATTGCTTGCATTTT  R: AACTGCCCTACAAGAGGGGAAAAGC | N/A | N/A | **14** | **N/A** | N/A | **91-129** | **16** | **0.807** | **0.842** | **0.043** | (Jahnke, et al. 2011, Zarouri 2016) |
| **VMCNG2B7.2** | F: TTTTGGAGTGAATAGAGACCCCT  R: CAGAATTTGGCTCCATATTTGAA | N/A | N/A | **14** | **N/A** | (GA)_13_ | **134-156** | **N/A** | **N/A** | **N/A** | **N/A** | (Arroyo-García and Martínez-Zapater 2004) |
| **VMCNG2E8** | F: CAGAGACAAAGGAAACGAGGCT  R: TGCCTACCTAGTGCCATTCAAA | N/A | N/A | **13** | **N/A** | (GA)_29_ | **190-208** | **N/A** | **N/A** | **N/A** | **N/A** | (Arroyo-García and Martínez-Zapater 2004) |
| **VMCNG2G7** | F: CAACAGAATTCAAATGAAATGGA  R: CAAACAGCATAAATACACAAGGA | 1:NC_012007.3 | 1:F 6993233- 6993255  R 6993332- 6993310 | **1** | **N/A** | (TC)_18_(TC)_7_ | **102-150** | **13** | **0.826** | **0.802** | **0.058** | (Arroyo-García and Martínez-Zapater 2004, Zarouri 2016) |
| **VMCNG2H7** | F: ACGTTAAATAGAACATGGTCCC  R: CAACCTCTTTTTTTGAGGTAGC | 1:NC_012018.3  2:NC_048442.1 | 1: F 15917119 -15917140  R 15917287 - 15917266  2: F 7448476- 7448455  R 7448305- 7448326 | **12** | **12** | (GA)_16_ | **150-178** | **N/A** | **N/A** | **N/A** | **N/A** | (Arroyo-García and Martínez-Zapater 2004) |
| **VrZAG12** | F: CTGCAAATAAATATTAAAAAATTCG  R: AAATCCTCGGTCTCTAGCCAAAAGG | N/A | Ν/Α | **15** | **Ν/Α** | (GA)_24_ | **140–172** | **N/A** | **>0.26** | **0.77** | **N/A** | (Sefc, Regner et al. 1999) |
| **VrZAG14** | F: ATCAAAGGCCTCCTTTATTGCCATC  R: TAGTACCAACTACCAACAACCAAAG | 1:NW_003724191.1 | 1: F 897819- 897843  R 897956- 897932 | **18** | **Ν/Α** | (A)_12_(GA)_16_ | **137–162** | **N/A** | **>0.21** | **0.52** | **N/A** | (Sefc, et al. 1999) |
| **VrZAG15** | F: GGATTTTGGCTGTAGTTTTGTGAAG  R: ATCTCAAGCTGGGCTGTATTACAAT | 1:NC_012023.3  2:NC_048447.1 | 1: F 6457122- 6457098  R 6456959- 6456982  2: F 13126445- 13126469  R 13126626- 13126603 | **17** | **17** | (GA)_19_ | **163–193** | **11** | **0.46** | **0.44** | **0.36** | (Sefc, et al. 1999, Zarouri 2016) |
| **VrZag21** | F: TCATTCACTCACTGCATTCATCGGC  R: GGGGCTACTCCAAAGTCAGTTCTTG | 1:NC_012010.3  2:NC_048434.1 | 1: F 13648595- 13648619  R 13648800- 13648776  2: F 13667960- 13667984  R 13668169- 13668145 | **4** | **4** | (GA)_16_ | **190–214** | **8** | **0.7162** | **0.7300** | **0.205** | (Sefc, et al. 1999, Hvarleva, et al. 2004, Jahnke, et al. 2011) |
| **VrZag25** | F: CTCCACTTCACATCACATGGCATGC  R: CGGCCAACATTTACTCATCTCTCCC | N/A | Ν/Α | **Ν/Α** | **Ν/Α** | (GA)_11_C(AG)_3_GG(AG)_3_ | **225–245** | **N/A** | **N/A** | **N/A** | **0.24** | (Sefc, et al. 1999, Jahnke, et al. 2011) |
| **VrZAG29** | F: ATAACCAGGACAAGTTATTCAAGCC  R: ACCCAATTGACCATCTTTTATGCTG | 1:NC_012007.3  2:NC_048431.1 | 1: F 5286106- 5286130  R 5286217- 5286193  2: F 5403151- 5403175  R 5403282- 5403258 | **1** | **1** | (GA)_19_ | **105-119** | **5** | **0.338** | **0.359** | **0.439** | (Sefc, et al. 1999, Zarouri 2016) |
| **VrZAG47** | F: GGTCTGAATACATCCGTAAGTATAT  R: ACGGTGTGCTCTCATTGTCATTGAC | 1: NC_012011.3  2: NC_048435.1 | 1: F 4472195-4472171  R 4472037-4472061  2: F 4125673-4125649  R 4125489-4125513 | **5** | **5** | (GA)_15_(AA)(GA)_5_ | **155-174** | **8** | **0.8243** | **0.8023** | **0.122** | (Sefc, et al. 1999, Hvarleva, et al. 2004, Galbács, et al. 2009) |
| **VrZAG64** | F: TATGAAAGAAACCCAACYCGGCACG  R: TGCAATGTGGTCAGCCTTTGATGGG | N/A | N/A | **10** | **N/A** | (TC)_27_ | **112-197** | **10** | **0.8919** | **0.8074** | **0.111** | (Hvarleva, et al. 2004, Veres, et al. 2004, Halasz, et al. 2005, Dos Anjos 2013) |
| **VrZag67** | F: ACCTGGCCCGACTCCTCTTGTATGC  R: TCCTGCCGGCGATAACCAAGCTATG | 1:NC_012016.3  2:NC_048440.1 | 1: F 1447885-1447861  R 1447733-1447757  2: F 17288150-17288174  R 17288306-17288282 | **10** | **10** | (GA)_18_(TA)_10_ | **126–159** | **17** | **0,796** | **0,829** | **0.09** | (Sefc, et al. 1999, Merkouropoulos, et al. 2015) |
| **VrZAG7** | F: GTGGTAGTGGGTGTGAACGGAGTGG  R: AACAGCATGACATCCACCTCAACGG | 2:NC_048440.1 | 2: F 498496-498472  R 498301-498325 | **N/A** | **10** | (AG)_4_T(AG)_21_ | **106–158** |  | **0.39** | **0.37** | **0.48** | (Sefc, et al. 1999) |
| **VrZAG83** | F: GGCGGAGGCGGTAGATGAGAGGGCG  R: ACGCAACGGCTAGTAAATACAACGG | 2:NC_048434.1 | 2: F 20847826-20847802  R 20847643-20847667 | **N/A** | **4** | (GA)_5_C(AG)_2_T(GA)_3_GG(GA)_2_T(AG)_7_ | **191-214** | **6** | **0.6081** | **0.7047** | **0.253** | (Sefc, et al. 1999, Hvarleva, et al. 2004, Galbács, et al. 2009, Bibi, et al. 2020) |
| **VVIb01** | F: TGACCCTCGACCTTAAAATCTT  R: TGGTGAGTGCAATGATAGTAGA | 1: NC_012008.3  2: NC_048432.1 | 1: F2349171-2349192  R2349460-2349439  2: F 17441702-17441681  R17441405-17441426 | **2** | **2** | (CT)_12_ | **250-300** | **4** | **0.80** | **0.70** | **0.575** | (Laucou, et al. 2011, Nebish, et al. 2017) |
| **VVIb09** | F: ATGTTTTGATTCCTTAGGTGAC  R: CCTAAGAGCCATTCAAGATTAA | 1:NC_012023.3  2:NC_048447.1 | 1: F 9295347-9295368  R 9295626-9295605  2: F 10473528-10473507  R 10473259-10473280 | **17** | **17** | (GT)_10_/(TC)_11_ | **250-300** | **5** | **0.80** | **0.78** | **N/A** | (Merdinoglu, et al. 2005) |
| **VVIb10** | F: CAAATCCTGAAAATGGCCTCAT  R: GAGCCCTTAAAACACTTTGACT | 1: NC_012018.3  2: NC_048442.1 | 1: F 18063282-18063261  R 18063207-18063228  F 18063282-18063261  R 18062999-18063020  2: F 5852597-5852618  R 5852668-5852647 | **12** | **12** | (GA)_12_ | **50-100** | **3** | **0.40** | **0.34** | **N/A** | (Merdinoglu, et al. 2005) |
| **VVIb18** | F: TTTTGTTTGCGAGGTTGGGATT  R: AATCAATCAACCACGCATGCTT | 1: NC_012012.3  2: NC_048436. | 1: F 6330751-6330730  R 6330570-6330591  2: F 16606767-16606788  R 16606948-16606927 | **6** | **6** | (GGT-GT)_3_-GG-(GT)_5_-CT-(GT)_3_ | **150-200** | **1** | **0.00** | **0.00** | **N/A** | (Merdinoglu, et al. 2005) |
| **VVIb19** | F: TGGATGTTCCTAAACCTTAAGT  R: GCATAAGGGCATTTTGGTAAAT | 1: NC_012017.3 | 1: F 5848909-5848930  R 5849300-5849279 | **11** | **N/A** | (CT)_6_-CG-(CT)_4_ | **350-400** | **2** | **0.00** | **0.18** | **N/A** | (Merdinoglu, et al. 2005) |
| **VVIb22** | F: CCCTCCAATCTACATCCATGAA  R: CAGTGTGTTTCTTGATGGTCCA | 1: NC_012013.3  2: NC_048437.1 | 1: F 3139481-3139502  R 3139640-3139619  2: F 27274422-27274401  R 27274277-27274298 | **7** | **7** | (CT)_8_-CA-(6CA-TA)_3_ | **150-200** | **3** | **0.60** | **0.46** | **N/A** | (Merdinoglu, et al. 2005) |
| **VVIb23** | F: GGTCACGTAGATATTGAAGTTG  R: TTTGTATTTTGGGCATTTGCAG | 1: NC_012008.3  2: NC_048432.1 | 1: F 4864625-4864604  R 4864334-4864355  2: F 14679430-14679451  R 14679740-14679719 | **2** | **2** | (GA)_12_ | **300-305** | **4** | **0.60** | **0.72** | **N/A** | (Merdinoglu, et al. 2005) |
| **VVIb31** | F: GGTTGGTACCAATGAAATCAAT  R: ACGTTCTCACAGTATTTCTCAA | 1: NC_012024.3  2: NC_048448.1 | 1: F 2913110-2913089  R 2912731-2912752  2: F 2940815-2940794  R 2940451-2940472 | **18** | **18** | (GA)_3_/(GA)_4_-AA-(GA)_9_-(GGA)_5_ | **350-400** | **3** | **0.40** | **0.46** | **N/A** | (Merdinoglu, et al. 2005) |
| **VVIb32** | F: GTAACCATCTCTAACCATTTCA  R: TGAGAACACTTCACAGAGATTT | 1: NC_012018.3  2: NC_048442.1 | 1: F 10307595-10307616  R 10307763-10307742  2: F 13864770-13864749  R 13864618-13864639 | **12** | **12** | (TG)_16_-(TA)_2_ | **150-200** | **6** | **0.60** | **0.82** | **N/A** | (Merdinoglu, et al. 2005) |
| **VVIb54** | F: GTCAAACATACATGCACCAACA  R: ACCAATGAAACCTAAAAGAGGG | 1: NC_012025.3  2: NC_048449.1 | 1: F 20082012-20081991  R 20081815-20081836  2: F 20472639-20472618  R 20472437-20472458 | **19** | **19** | (CA)_4_/(CA)_5_ | **150-200** | **1** | **0.00** | **0.00** | **N/A** | (Merdinoglu, et al. 2005) |
| **VVIb59** | F: ATCATTCTGATCCAGCTAATCC  R: TTCCTGAGTTGCTTTCATTAGC | 1: NC_012009.3  2:N/A | 1: F 8121467-8121446  R 8121111-8121132 | **3** | **N/A** | (GA)_30_ | **350-400** | **4** | **0.60** | **0.64** | **N/A** | (Merdinoglu, et al. 2005) |
| **VVIb63** | F: ACCACCAACATATATAGTCCAA  R: GAGAGAAATGTGGAGGAGTAAA | 1: NC_012021.3  2: NC_048445.1 | 1: F 11597299-11597278  R 11597153-11597174  2: F 9459483-9459504  R 9459637-9459616 | **15** | **15** | (GA)_13_/(TA)_2_-(GA)_4_ | **150-200** | **4** | **0.80** | **0.66** | **N/A** | (Merdinoglu, et al. 2005) |
| **VVIb66** | F: CCACTAGTGGTCAGAAAAGAAG  R: TTGTATTGTGTGCCTCTTCTCA | 1: NC_012014.3  2: NC_048438.1 | 1: F 18270347-18270326  R 18270246-18270267  2: F 18444409-18444388  R 18444322-18444343 | **8** | **8** | (GA)_13_ | **50-100** | **8** | **1.00** | **0.86** | **N/A** | (Merdinoglu, et al. 2005) |
| **VVIb68** | F: AATACATACATCCCATAAGGAG  R: TTGTGATGCAATACTCGTTGAT | 1: NC_012022.3  2: NC_048446.1 | 1: F 15787960-15787939  R 15787783-15787804  2: F 16377363-16377342  R 16377190-16377211 | **16** | **16** | (GA)_4_/(GA)_9_ | **150-200** | **3** | **0.80** | **0.54** | **N/A** | (Merdinoglu, et al. 2005) |
| **VVIb72** | F: TCAACTTAATTTCCTGATCCGA  R: CCAATTGATGGAGTATACTCAT | 1: NC_012011.3  2: NC_048435.1 | 1: F 2848403-2848424  R 2848585-2848564  2: F 2354282-2354303  R 2354464-2354443 | **5** | **5** | (CA)_3_/(CA)_6_ | **150-200** | **2** | **0.20** | **0.18** | **N/A** | (Merdinoglu, et al. 2005) |
| **VVIb94** | F: AGAAATCACATGAGAAAGCTGT  R: ATTCACTTCTCCAAACGCTTTT | 1: NC_012007.3  2: NC_048431.1 | 1: F 6835061-6835040  R 6834767-6834788  2: F 6880258-6880237  R 6879970-6879991 | **1** | **1** | (CT)_12_ | **300-350** | **4** | **0.80** | **0.66** | **N/A** | (Merdinoglu, et al. 2005) |
| **VVIc05** | F: GCGATTAAGCAAGTTGAAGAAC  R: AATGATTGCAAATAGATAGGGC | 1: NC_012017.3  2:N/A | 1: F 13520289-13520310  R 13520468-13520447 | **11** | **N/A** | (CT)_12_ | **150-200** | **2** | **0.00** | **0.32** | **N/A** | (Merdinoglu, et al. 2005) |
| **VVIc35** | F: GGAAAAATGATGAGGTAAAGCC  R: GGCAGATATGGAGAAACAAATG | 1: NC_012021.3  2: NC_048445.1 | 1: F9660794-9660815  R9661165-9661144  2: F11458399-11458378  R11458039-11458060 | **15** | **15** | (TC)_25_ | **350-400** | **4** | **0.40** | **0.56** | **N/A** | (Merdinoglu, et al. 2005) |
| **VVIc46** | F: TTGCAACATGGATTCCCTTTTT  R: AAATGGATTTTTGTGGGGGAAT | 1: NC_012014.3  2: NC_048438.1 | 1: F 15047468-15047447  R 15047378-15047399  2: F 15103858-15103837  R 15103768-15103789 | **8** | **8** | (TCA)_5_ | **50-100** | **2** | **0.20** | **0.18** | **N/A** | (Merdinoglu, et al. 2005) |
| **VVIc50** | F: TTGTTAGCCACAATTCAAGAGG  R: TGTTATGGACAAGATGAAAGGC | 1: NC_012012.3  2: NC_048436.1 | 1: F 8450955-8450976  R 8451033-8451012  2: F 14311014-14310993  R 14310928-14310949 | **6** | **6** | (GA)_12_ | **50-100** | **4** | **0.20** | **0.58** | **N/A** | (Merdinoglu, et al. 2005) |
| **VVIc51** | F: CTTTGAAGCACAAAATCGAGCT  R: ACCAAAGGGAAGCAAAAGAAAA | 1: NC_012019.3  2: NC_048443.1 | 1: F 8083860-8083839  R 8083696-8083717  2: F 20214060-20214081  R 20214223-20214202 | **13** | **13** | (CTTCT)_3_-(CT)_11_ | **150-200** | **3** | **0.60** | **0.46** | **N/A** | (Merdinoglu, et al. 2005) |
| **VVIc72** | F: GTATTGTGTAAGCATTGTGTGG  R: GGACAAGGAGTTAGATATGAAC | 1: NC_012007.3  2: NC_048431.1 | 1: F 3255985-3255964  R 3255733-3255754  2: F 3261527-3261506  R 3261273-3261294 | **1** | **1** | (AG)_5_-GGAA-(AG)_3_-GG-(AG)_8_ | **300-350** | **3** | **0.80** | **0.54** | **N/A** | (Merdinoglu, et al. 2005) |
| **VVIf52** | F: AGGGAATTGAAGAGAAACTGTT  R: TCTGCCAAGCAAATGAAAGAAA | 1: NC_012007.3  2: NC_048431.1 | 1: F 22273039-22273060  R 22273297-22273276  2: F 23238182-23238203  R 23238432-23238411 | **1** | **1** | (AG)_3_-GAG-(GGA)_2_-(GA)_2_-(GG-3AG)_3_-(AG)_3_-(GGAA)_2_/(TG)_4_ | **300-350** | **5** | **0.80** | **0.83** | **N/A** | (Merdinoglu, et al. 2005) |
| **VVIh01** | F: GGGCTTTGCTGCGATATTTATT  R: ACACAGAATACGCAACTTTGCA | 1: NC_012016.3  2: NC_048440.1 | 1: F 1181498-1181477  R 1181256-1181277  2: F 21645480-21645501  R 21645719-21645698 | **10** | **10** | (CT)_20_ | **300-350** | **5** | **0.80** | **0.76** | **N/A** | (Merdinoglu, et al. 2005) |
| **VVIh02** | F: AGAACACTTTGGTAAGAGGCAA  R: TTCTATACGACGTAGCCCAAAA | 1: NC_012009.3  1: NC_012015.3  2: NC_048439.1 | 1: F 9073477-9073456  R 9073342-9073363  F 703090-703069  R 702969-702990  2: F 710088-710067  R 709965-709986 | **3, 9** | **9** | (TC)_12_ | **50-100** | **4** | **1.00** | **0.70** | **N/A** | (Merdinoglu, et al. 2005) |
| **VVIh54** | F: CCGCACTTGTGTTGAATTTCAG  R: CAAACCGTTTTTACACCAGCAG | 1: NC_012019.3  2: NC_048443.1 | 1: F 3333487-3333508  R 3333652-3333631  2: F 25894227-25894206  R 25894062-25894083 | **13** | **13** | (GA)_18_ | **150-200** | **4** | **0.80** | **0.66** | **0.828** | (Merdinoglu, et al. 2005; Laucou, et al., 2011; Nebish, et al. 2017) |
| **VVIi51** | F: ATCCCAAGAGAACCAAGAAACT  R: GCTGATCTCAGTGCATATGTTG | 1: NC_012020.3  2: NC_048444.1 | 1: F 28772163-28772184  R 28772424-28772403  2: F 1550482-1550461  R 1550235-1550256 | **14** | **14** | (GA)_17_ | **300-350** | **5** | **1.00** | **0.76** | **N/A** | (Merdinoglu, et al. 2005) |
| **VVIi52** | F: AGATTTAGAGACGAAAAAGGGT  R: CTTGATCTTTAGTTGCAGTCTG | 1: NC_012011.3  2: NC_048435.1 | 1: F 4629717-4629696  R 4629620-4629641  2: F 4247694-4247673  R 4247605-4247626 | **5** | **5** | (GT)_12_ | **50-100** | **3** | **0.40** | **0.58** | **N/A** | (Merdinoglu, et al. 2005) |
| **VVIm01** | F: GAAGAATTTTAGGAGTTGGTCA  R: GAAGAGAAGCAAGAAGTGATAA | 1: NC_012019.3  2: N/A | 1: F 6508124-6508145  R 6508306-6508285  2: F N/A  R N/A | **13** | **N/A** | (CA)_3_-GA-(CA)_9_ | **150-200** | **2** | **0.40** | **0.32** | **N/A** | (Merdinoglu, et al. 2005) |
| **VVIm03** | F: ACTTTGCACTTCCCCTTAAAAA  R: ATGGATATGCTGATAGTGATGT | 1: NC_012025.3  2: NC_048449.1 | 1: F 7078556-7078577  R 7078930-7078909  2: F 7515415-7515436  R 7515787-7515766 | **19** | **19** | (GA)_12_ | **350-400** | **4** | **0.40** | **0.58** | **N/A** | (Merdinoglu, et al. 2005) |
| **VVIm04** | F: AAAAATCTGATATCAACCGGCT  R: TATATTGATCACCACACTCGAT | 1: NC_012017.3  2: NC_048441.1 | 1: F 558439-558460  R 558521-558500  2: F 19307340-19307319  R 19307250-19307271 | **11** | **11** | (GA)_14_ | **50-100** | **5** | **0.60** | **0.68** | **N/A** | (Merdinoglu, et al. 2005) |
| **VVIm07** | F: TGGTGTCAACATTCCTTACAAG  R: TTATTACATGGATAGGCACTCA | 1: NC_012014.3  2: NC_048438.1 | 1: F 10702981-10703002  R 10703329-10703308  2: F 10787839-10787860  R 10788195-10788174 | **8** | **8** | (TC)_5_-TA-(TC)_14_ | **350-400** | **3** | **0.80** | **0.64** | **N/A** | (Merdinoglu, et al. 2005) |
| **VVIm10** | F: AGGTGAACTCTGTAAATATACG  R: GCTCAAAGTTGAAGATTTATCC | 1: NC_012024.3  2: NC_048448.1 | 1: F 10439563-10439542  R 10439196-10439217  2: F 10689192-10689171  R 10688827-10688848 | **18** | **18** | (GT)_8_-(GA)_8_ | **350-400** | **4** | **0.80** | **0.58** | **N/A** | (Merdinoglu, et al. 2005) |
| **VVIM10** | F: AAAAGGTGAACTCTGTAAATATACG  R: GGATAAATCTTCAACTTTGAGCAGC | 1: N/A  2: N/A | 1: N/A  2: N/A | **18** | **N/A** | (GT)_8_-(GA)_8_ | **350-400** | **4** | **0.80** | **0.58** | **N/A** | (Merdinoglu, et al. 2005, Jahnke, et al. 2011) |
| **VVIm11** | F: AAAAGCCCATTAAGTGCCAATG  R: CCTATGAACTTATTGGGCTCTT | 1: NC_012018.3  2: NC_048442.1 | 1: F8350835-8350856  R8351130-8351109  2: F12338916-12338895  R12338637-12338658 | **12** | **12** | (CT)_8_-(TC)_8_ | **300-350** | **4** | **0.80** | **0.74** | **N/A** | (Merdinoglu, et al. 2005) |
| **VVIm25** | F: TGTTTTAACAGAAGCCTACACG  R: GAGAGTGATGTGGGATTTGTTA | 1: NC_012007.3  2: NC_048431.1 | 1: F 12224157-12224178  R 12224323-12224302  2: F 13237272-13237293  R 13237440-13237419 | **1** | **1** | (CT)_12_ | **150-200** | **5** | **0.80** | **0.68** | **N/A** | (Merdinoglu, et al. 2005) |
| **VVIm26** | F: CTCACCCTTGGTGTTGAAGTTG  R: CGTCACTCACCTCCAAAGCTTC | 1:NW_003724290.1  2: NC_048437.1 | 1: F 21599-21620  R 21765-21744  2: F 7574793-7574772  R 7574627-7574648 | **N/A** | **7** | (AG)_5_/(AG)_3_ | **150-200** | **1** | **0.00** | **0.00** | **N/A** | (Merdinoglu, et al. 2005) |
| **VVIm33** | F: CTGAACCTGAAACTGATGAAGT  R: ATGAATGGACAGTGCAACTTTG | 1: NC_012013.3  2: NC_048448.1 | 1: F 5667762-5667783,  R 5667881-5667860  2: F 38950751-38950730, R 38950475-38950496 | **18** | **18** | (GA)_10_-(CA)_7_-(CA)_6_ | **300-350** | **1** | **0.00** | **0.00** | **N/A** | (Merdinoglu, et al. 2005) |
| **VVIm42a** | F: AATGGTGGTAAAGTCTTTGCTG  R: TCTTCCAATAATACCAGCACTG | 1: NC_012021.3  2: NC_048445.1 | 1:F 15943609-15943630  R 15943863-15943842  2:F 4747445-4747424  R 4747173-4747194 | **15** | **15** | (ACC)_13_-(AAC)_6_/(AG)_3_ | **300-350** | **4** | **0.40** | **0.48** | **N/A** | (Merdinoglu, et al. 2005) |
| **VVIm42b** | F: CCCTCAAGACCTTGAAAATTGT  R: ACACAAATATGCATACACACGC | 1: NC_012021.3  2: NC_048445.1 | 1:F 15943274-15943295  R 15943358-15943337  2:F 4747783-4747762  R 4747699-4747720 | **15** | **15** | (GT)_7_ | **50-100** | **3** | **0.60** | **0.46** | **N/A** | (Merdinoglu, et al. 2005) |
| **VVIm43** | F: GGTGTTGTTTTCTTGTGTTTGT  R: AGGATACATGCTGAAGAATATG | 1: NC_012012.3  2: NC_048436.1 | 1:F 19003995-19003974  R 19003918-19003939  2:F 2854868-2854889  R 2854953-2854932 | **6** | **6** | (TC)_17_ | **50-100** | **5** | **0.80** | **0.76** | **N/A** | (Merdinoglu, et al. 2005) |
| **VVIm58** | F: ACAATTAGTATCAAAGCCAACC  R: TTGGGTTGATCAACACTTGTTT | 1: NC_012012.3  2: NC_048443.1 | 1:F 8025477- 8025498  R 8025863 - 8025842  2:F 769723- 769702  R 769360- 769381 | **6** | **13** | (AT)_4_/(AT)_3_-(GT)_5_-AC-(GT)_6_ | **350-400** | **1** | **0.00** | **0.00** | **N/A** | (Merdinoglu, et al. 2005) |
| **VVIm63** | F: AAGCCTTCTACTTGTTTGATGA  R: ATTTGTTCTCAAACAGGCACAT | 1: NC_012019.3  2: NC_048443.1 | 1:F 4808159-4808138  R 4807861-4807882  2:F 24394214-24394235  R 24394520-24394499 | **13** | **13** | (TC)_4_-TATC-(TG)_3_ | **300-350** | **2** | **0.20** | **0.18** | **N/A** | (Merdinoglu, et al. 2005) |
| **VVIm72** | F: AATGTCTTACAGCACTATTTGG  R: TGAAGATGATATACAGAGTAGC | 1: NC_012024.3  2: NC_048448.1 | 1:F 6009889-6009868  R 6009558-6009579  2:F 6048058-6048037  R 6047737-6047758 | **18** | **18** | (CAA)_3_-AT-(GA)_18_ | **350-400** | **1** | **0.00** | **0.00** | **N/A** | (Merdinoglu, et al. 2005) |
| **VVIm79a** | F: ATTATGTTTGAAGACTTGTGCC  R: GTGAGATCTCTAGAGAAGTTTG | 1: NC_012024.3  2: NC_048448.1 | 1: F 13555077-13555056  R 13554857-13554878  2: 13759781-13759760  R 13759561-13759582 | **18** | **18** | (TTTTC)_2_ | **150-200** | **1** | **0.00** | **0.00** | **N/A** | (Merdinoglu, et al. 2005) |
| **VVIm79b** | F: AAACTTCTCTAGAGATCTCACC  R: AGTGTGTTTGACTCCAGAAAAG | 1: N/A  2: N/A | 1: N/A  2: N/A | **N/A** | **N/A** | (TC)_9_-TT-(TC)_10_-(T)_13_-C-(T)_6_ | **300-350** | **5** | **0.20** | **0.74** | **N/A** | (Merdinoglu, et al. 2005) |
| **VVIm93** | F: CAACGTTTATTGTAAGAGCCTC  R: GCTTAGCTTGCTAGAAACTTGA | 1: NC_012024.3  2: NC_048448.1 | 1: F 6962396-6962375  R 6962288-6962309  2: F 6934454-6934433  R 6934344-6934365 | **18** | **18** | (CCCT)_4_-CCC-(CCCT)_2_-(CT)_3_ | **150-200** | **4** | **0.40** | **0.56** | **N/A** | (Merdinoglu, et al. 2005) |
| **VVIn03** | F: ACTCAAATTTGTTCCCTACTCT  R: TGAGAAAATCTGAACCAGACTA | 1: NC_012022.3  2: NC_048446.1 | 1: F 388101-388122  R 388445-388424  2: F 342239-342260  R 342585-342564 | **16** | **16** | (AC)_3_-(TAA)_2_/(TG)_3_-GG-TT-(TG)_2_ | **350-400** | **1** | **0.00** | **0.00** | **N/A** | (Merdinoglu, et al. 2005) |
| **VVIn04** | F: GTGAAGGTGGTGTTTGTAGATT  R: CCTAATTGGCTGAATGTGATCT | 1: NC_012025.3  2: NC_048449.1 | 1: F 6696956-6696977  R 6697317-6697296  2: F 7087084-7087105  R 7087444-7087423 | **19** | **19** | TT-(CT)_5_/(TA)_2_-(GA)_2_ | **350-400** | **4** | **0.60** | **0.64** | **N/A** | (Merdinoglu, et al. 2005) |
| **VVIn16** | F: ACCTCTATAAGATCCTAACCTG  R: AAGGGAGTGTGACTGATATTTC | 1: NC_012024.3  2: NC_048448.1 | 1: F 23389846-23389825,  R 23389686-23389707  2: F 29558337- 29558316,  R 29558187-29558208 | **18** | **18** | (AC)_3_-G-(CA)_6_ | **150-200** | **3** | **0.80** | **0.62** | **0.628** | (Merdinoglu, et al. 2005, Laucou, et al. 2011, Nebish, et al. 2017) |
| **VVIn31** | F: GTTGAATAGTGTCCATGTTGTG  R: GGATAGAATCACATTTGTAGCG | 1: NC_012012.3 | 1: F 17800616- 17800595,  R 17800420- 17800441 | **6** | **N/A** | (AGAT)4-(3(AG)-AT)_4_-(AG)_14_-(AT)_2_ | **150-200** | **6** | **0.80** | **0.76** | **N/A** | (Merdinoglu, et al. 2005) |
| **VVIn33** | F: TGCCAAAGCAAGTATCAACATG  R: ATTTTGATCCCACCTAACTCTG | 1: NC_012011.3  2: NC_048435.1 | 1: F 11299447-11299468  R 11299723- 11299702  2: F 11510823-11510844  R 11511081- 11511060 | **5** | **5** | (TC)_4_-CC-AC-(TC)_10_-AC-(TC)_8_-AC-GC-(AC)_5_-(TC)_2_ | **300-350** | **4** | **0.80** | **0.74** | **N/A** | (Merdinoglu, et al. 2005) |
| **VVIn73** | F: TACTTCACCTAACAATACAGCT  R: AATACATAAGGTGAAGATGCCT | 1: NC_012023.3  2: NC_048447.1 | 1: F 5504674 - 5504653  R 5504409 - 5504430  2: F 14035715- 14035736  R 14035970- 14035949 | **17** | **17** | (AC)_10_ | **260–272** | **5** | **0.595** | **0.514** | **0.459** | (Merdinoglu, et al. 2005, Laucou, et al. 2011, Nebish, et al. 2017) |
| **VVIp31** | F: TATCCAAGAGACAAATTCCCAC  R: TTCTCTTGTTTCCTGCAAATGG | 1: NC_012025.3  2: N/A | 1: F 6697524 - 6697545  R 6697705 - 6697684  2: N/A | **19** | **N/A** | (GA)_20_ | **173–196** | **11** | **0.895** | **0.847** | **0.818** | (Merdinoglu, et al. 2005, Laucou, et al. 2011, Nebish, et al. 2017) |
| **VVIp60** | F: GGGGAATAACTAAATTGAGGAT  R: GTATGAATGCGGATAGTTTGTG | 1: NC_012007.3  2: NC_048431.1 | 1: F 8803413-8803434  R 8803728-8803707  2 F 9853301-9853322  R: 9853622-9853601 | **1** | **1** | (TG)_8_-AG-(TG)_10_-(AG)_12_ | **306–332** | **9** | **0.789** | **0.755** | **0.710** | (Merdinoglu, et al. 2005, Laucou, et al. 2011, Nebish, et al. 2017) |
| **VVIQ52** | F: TAAAAGGATGGTAGATGACAGA  R: ACAGGAAAGTGTTCAATGGTTA | 1: NC_012015.3  2: NC_048439.1 | 1: F 21558125-21558104  R 21558043-21558064  2: F 22143424-22143403  R 22143332-22143353 | **9** | **9** | (AC)_2_-TC)_11_ | **82-93** | **5** | **0.686** | **0.667** | **0.173** | (Merdinoglu, et al. 2005, Zarouri 2016) |
| **VVIV37** | F: TTTTCTCCCTACTCTTAACTTC  R: GGTAGACCTTGAAATGAAGTAA | 1: NC_012016.3  2: NC_048440.1 | 1:F 11032502-11032481  R 11032334-11032355  2:F 8762741-8762762  R 8762899-8762878 | **10** | **10** | (TC)_16_-(TG)_3_ | **150-200** | **4** | **0.80** | **0.64** | **0.760** | (Merdinoglu, et al. 2005, Nebish, et al. 2017) |
| **VVIV67** | F: TATAACTTCTCATAGGGTTTCC  R: TTGGAGTCCATCAAATTCATCT | 1: NC_012021.3  2: NC_048445.1 | 1: F 10897758-10897737  R 10897392-10897413  2: F 17911479-17911500  R 17911867-17911846 | **15** | **15** | (CA)_3_-AT-(CA)_2_-(GA)_4_-TT-(GA)_2_/(AG)_15_ | **350-400** | **5** | **1.00** | **0.74** | **0.777** | (Merdinoglu, et al. 2005, Nebish, et al. 2017) |
| **VVMD15** | F: CTGCAGTGCACTCAAAGTTGG  R: TGAAACACCAAGGGAAACCTC | 1: N/A  2: N/A | 1: N/A  2: N/A | **N/A** | **N/A** | (GA)_19_ | **192–216** | **12** | **0.95** | **0.86** | **0.845** | (Sefc, et al. 1999, Jing, et al. 2013, Žulj , Maletić et al. 2020) |
| **VVMD19** | F: TGAAATATCATCAATGCTCTCTCTCC  R: GGTTGATATTGCTTCCTTTTCCC | 1:NW_003724213.1  2: NC_048440.1 | 1:F 194851-194876  R195046 -195024  2:F 21094485-21094460  R: 21094290-21094312 | **N/A** | **10** | N/A | **N/A** | **N/A** | **N/A** | **N/A** | **N/A** | (Jing, et al. 2013) |
| **VVMD21** | F: GGTTGTCTATGGAGTTGATGTTGC  R: GCTTCAGTAAAAAGGGATTGCG | 1: NC_012012.3  2: NC_048436.1 | 1:F 13758808-13758831  R 13759052-13759032  2: F 7531145-7531122  R 7530928-7530948 | **6** | **6** | (A)_13_(A)_21_(T)_18_(C)_9_(T)_22_(AT)_6_ | **230-267** | **7** | **0.678** | **0.707** | **0.219** | (Galbács, et al. 2009, Dos Anjos 2013) |
| **VVMD24** | F: GTGGATGATGGAGTAGTCACGC  R: GATTTTAGGTTCATGTTGGTGAAGG | 1:NC_012020.3  2:NC_048444.1 | 1: F 24252077-24252056  R 24251864-24251888  2:F 6056744-6056765  R 6056947-6056923 | **14** | **14** | (CT)_11_(T)_10_(A)_10_ | **206–219** | **7** | **0.868** | **0.809** | **0.770** | (Laucou, et al. 2011, Dos Anjos 2013, Nebish, et al. 2017) |
| **VVMD36** | F: GAAAATTAATAGGGGGACACGGG  R: GCAACTGTAAAGGTAAGACACAGTCC | 1:NC_012009.3  2:NC_048433.1 | 1: F 7904163-7904185  R 7904412-7904387  2: F 7525800-7525822  R 7526047-7526022 | **3** | **3** | N/A | **244-296** | **17** | **0.817** | **0.838** | **0.082** | (Halasz, et al. 2005, Galbács, et al. 2009) |
| **VVMD6** | F: ATCTCTAACCCTAAAACCAT  R: CTGTGCTAAGACGAAGAAGA | 1:NC_012013.3  2:NC_048437.1 | 1: F 4186801-4186820  R 4187000-4186981  2: F 26216152-26216133  R 26215948-26215967 | **7** | **7** | (CT)C(CT)TTAG(CT)TAAT-(CT)_6_C(CT)_2_C(CT)_2_ | **194-214** | **5** | **N/A** | **N/A** | **N/A** | (Bowers, et al. 1996) |
| **VVMD8** | F: TAACAAACAAGAAGAGGAAT  R: AGCACATCCACAACATAATG | 1:NC_012017.3  2:NC_048441.1 | 1: F 19674992-19675011  R 19675145-19675126  2: F 159556-159537  R 159376-159395 | **11** | **11** | (TC)_12.5_(TA)_8_ | **131-215** | **23** | **0.754** | **0.798** | **0.066** | (Bowers, et al. 1996, Zarouri 2016) |
| **VVS1** | F: ACAATTGGAAACCGCGTGGAG  R: CTTCTCAATGATATCTAAAACCATG | 1: NC_012019.3  2: NC_048443.1 | 1:F 6563366 - 6563346  R 6563177 - 6563201  2:F 22468337-22468357  R 22468519 - 22468495 | **13** | **13** | (AG)_15_ | **160-205** | **5** | **N/A** | **N/A** | **N/A** | (Thomas and Scott 1993, Lefort and Roubelakis-Aggelakis 2000, Dos Anjos 2013; Dauob, et al. 2018) |
| **VvUCH29** | F: AAACATGATCTGATGCAGGTGA  R: CAACCTGTTGATGAAAGGGAAA | 1: AF143277 | N/A | **N/A** | **N/A** | (CT)_18_ | **207-315** | **26** | **0.793** | **0.845** | **N/A** | (Lefort, et al. 2002) |

**Reference list**

Arroyo-García, R. and J. Martínez-Zapater (2004). “Development and characterization of new microsatellite markers for grape.” Vitis **43** (4): 175–178

Bibi, A. C., E. D. Gonias and A. G. Doulis (2020). “Genetic diversity and structure analysis assessed by SSR Markers in a large collection of *Vitis c*ultivars from the island of Crete, Greece.” Biochemical Genetics: **58**, 294–321.

Bowers, J. E., G. S. Dangl and C. P. Meredith (1999). “Development and Characterization of Additional Microsatellite DNA Markers for Grape.” American Journal of Enology and Viticulture **50**(3): 243.

Bowers, J. E., G. S. Dangl, R. Vignani and C. P. Meredith (1996). “Isolation and characterization of new polymorphic simple sequence repeat loci in grape (*Vitis vinifera* L.).” Genome **39**(4): 628-633.

Carimi, F., F. Mercati, L. Abbate and F. Sunseri (2010). “Microsatellite analyses for evaluation of genetic diversity among Sicilian grapevine cultivars.” Genetic Resources and Crop Evolution **57**(5): 703-719.

Cipriani, G., M. T. Marrazzo, G. Di Gaspero, A. Pfeiffer, M. Morgante and R. Testolin (2008). “A set of microsatellite markers with long core repeat optimized for grape (*Vitis spp*) genotyping.” BMC Plant Biology **8**(1): 127.

Dauob, R., G. Makhoul and H. Mahfoud (2018). “Genetic Diversity among Grapevine (*Vitis Vinifera* L) Cultivars of Tartous Province (Syria) using Microsatellite Markers.” International Journal of Agriculture & Environmental Science **5**: 54-58.

Dos Anjos, L. M. (2013). Genetic diversity of Plasmopara viticola and genetic mapping of downy mildew resistance QTLs in grapevine (*Vitis* Spp.), PhD Thesis, Universidade de Brasília, Brasil.

Drábek, J., M. Smolíková, R. Kalendar, F. A. L. Pinto, P. Pavloušek, K. Klepárník and I. Frébort (2016). “Design and validation of an STR hexaplex assay for DNA profiling of grapevine cultivars.” Electrophoresis **37**(23-24): 3059-3067.

Ekhvaia, J., M. Gurushidze, F. R. Blattner and M. Akhalkatsi (2014). “Genetic diversity of *Vitis* *vinifera* in Georgia: relationships between local cultivars and wild grapevine, V vinifera L subsp *sylvestris*.” Genetic Resources and Crop Evolution **61**(8): 1507-1521.

Galbács, Z., S. Molnár, G. Halász, P. Kozma, S. Hoffmann, L. Kovacs, A. Veres, Z. Galli, A. Szőke, L. Heszky and E. Kiss (2009). “Identification of grapevine cultivars using microsatellite-based DNA barcodes.” Vitis **48**.

Gaspero, G., E. Peterlunger, R. Testolin, K. Edwards and G. Cipriani (2000). “Conservation of microsatellite loci within the genus *Vitis*.” Theoretical and Applied Genetics **101**: 301-308.

Guo, D.-L., Y.-H. Yu, F.-F. Xi, Y.-Y. Shi and G.-H. Zhang (2016). “Histological and molecular characterization of grape early ripening bud mutant.” International Journal of Genomics **2016**.

Halasz, G., A. Veres, P. Kozma, E. Kiss, Balogh, Z. Galli, A. Szoke, S. Hoffmann and L. Heszky (2005). “Microsatellite fingerprinting of grapevine (*Vitis vinifera* L) varieties of the Carpathian Basin.” Vitis **44**(4): 173-180.

Hvarleva, T., K. Rusanov, F. Lefort, I. Tsvetkov, A. Atanassov and I. Atanassov (2004). “Genotyping of Bulgarian *Vitis vinifera* L. cultivars by microsatellite analysis.” Vitis **43**(1): 27-34.

Italian_Vitis_Database (2022). <https://vitisdb.it/descriptors/microsatellites>.

Jahnke, G., J. Májer, A. Lakatos, J. G. Molnár, E. Deák, É. Stefanovits-Bányai and P. Varga (2009). “Isoenzyme and microsatellite analysis of *Vitis vinifera* L. varieties from the Hungarian grape germplasm.” Scientia Horticulturae **120**(2): 213-221.

Jing, Z. B., X. P. Wang and J. M. Cheng (2013). “Analysis of genetic diversity among Chinese wild *Vitis* species revealed with SSR and SRAP markers.” Genet Mol Res **12**(2): 1962-1973.

Karatas, D. D., H. Karatas, V. Laucou, G. Sarikamis, L. Riahi, R. Bacilieri and P. This (2014). “Genetic diversity of wild and cultivated grapevine accessions from southeast Turkey.” Hereditas **151**(4-5): 73-80.

Laucou, V., T. Lacombe, F. Dechesne, J.-P. R. Siret, M. Bruno, T. Dessup, P. Ortigosa, P. Parra, C. Roux, S. Santoni, D. Vare`s, J.-P. Pe´ros, J.-M. Boursiquot and P. This (2011). “High throughput analysis of grape genetic diversity as a tool for germplasm collection management.” Theoretical and Applied Genetics (122): 1233-1245.

Lefort, F., C. J. Kyvelos, M. Zervou, K. J. Edwards and K. A. Roubelakis-Angelakis (2002). “Characterization of new microsatellite loci from *Vitis vinifera* and their conservation in some *Vitis* species and hybrids.” Molecular Ecology Notes **2**(1): 20-21.

Lefort, F. and K. Roubelakis-Aggelakis (2000). “The Greek *Vitis* Database: a multimedia web-backed geneticdatabase for germplasm management of *Vitis* resources in Greece.” Journal of Wine Research **11**(3):233–242. <http://greekvitisdbbiologyuocgr/>.

Marques da Silva, J., A. Figueiredo, J. Cunha, J. E. Eiras-Dias, S. Silva, L. Vanneschi and P. Mariano (2020). “Using rapid chlorophyll fluorescence transients to classify *Vitis* genotypes.” Plants **9**(2): 174.

Merdinoglu, D., G. Butterlin, L. Bevilacqua, V. Chiquet, A.-F. Adam-Blondon and S. Decroocq (2005). “Development and characterization of a large set of microsatellite markers in grapevine (*Vitis vinifera* L) suitable for multiplex PCR.” Molecular Breeding **15**(4): 349-366.

Merkouropoulos, G., S. Michailidou, A. Alifragkis, E. Zioziou, S. Koundouras, A. Argiriou and N. Nicolaou (2015). “A combined approach involving ampelographic description, berry oenological traits and molecular analysis to study native grapevine varieties of Greece.” Vitis **54**: 99-103.

Nebish, A., I. Ochssner, E. Maul, R. Töpfer, L. Hausmann, A. Hovhannisyan, H. Devejyan, G. Melyan and R. Aroutiounian (2017). “Genetic identification and characterization of Armenian grapevine cultivars.” BIO Web Conf **9**: 01020.

Rustioni, L., G. De Lorenzis, M. Harta and O. Failla (2016). “Pink berry grape (*Vitis vinifera* L) characterization: Reflectance spectroscopy, HPLC and molecular markers.” Plant Physiology and Biochemistry **98**: 138-145.

Schuck, M. R., F. M. Moreira, M. P. Guerra, J. A. Voltolini, M. S. Grando and A. L. d. Silva (2009). “Molecular characterization of grapevine from Santa Catarina, Brazil, using microsatellite markers.” Pesquisa Agropecuária Brasileira **44**: 487-495.

Scott, K. D., P. Eggler, G. Seaton, M. Rossetto, E. M. Ablett, L. S. Lee and R. J. Henry (2000). “Analysis of SSRs derived from grape ESTs.” Theoretical and Applied Genetics **100**(5): 723-726.

Sefc, K., I. Pejić, E. Maletić, M. Thomas and F. Lefort (2009). Microsatellite markers for grapevine: tools for cultivar identification & pedigree reconstruction. Grapevine molecular physiology & biotechnology, Springer**:** 565-596.

Sefc, K. M., F. Regner, E. Turetschek, J. Glössl and H. Steinkellner (1999). “Identification of microsatellite sequences in *Vitis riparia* and their applicability for genotyping of different *Vitis* species.” Genome **42**(3): 367-373.

Stavrakaki, M., D. Bouza and K. Biniari (2020). “Differentiation of Greek grapevine cultivars (*Vitis vinifera* L.) based on the combination of ampelographic description and microsatellite markers.” Genetic Resources and Crop Evolution **67**(1): 21-40.

Thomas, M. R. and N. S. Scott (1993). “Microsatellite repeats in grapevine reveal DNA polymorphisms when analysed as sequence-tagged sites (STSs).” Theoretical and Applied Genetics **86**(8): 985-990.

Veres, A., A. Balogh, E. Kiss, A. Szőke, L. Heszky, P. Kozma, M. Kocsis and Z. Galli (2004). "Characterization of grapevine cultivars autochthonous in the Carpathian basin with microsatellites." Acta Horticulturae: 467-470.

Wang, L., J. Zhang, L. Liu, L. Zhang, L. Wei and D. Hu (2015). "Genetic diversity of grape germplasm as revealed by microsatellite (SSR) markers." African Journal of Biotechnology **14** (12): 990-998.

Ye, J., G. Coulouris, I. Zaretskaya, I. Cutcutache, S. Rozen and T. L. Madden (2012). "Primer-BLAST: a tool to design target-specific primers for polymerase chain reaction." BMC Bioinformatics **13**(1): 1-11.

Zarouri, B. (2016). Association study of phenology, yield and quality related traits in table grapes using SSR and SNP markers. Doctoral, Universidad Politecnica de Madrid

Žulj Mihaljević, M., E. Maletić, D. Preiner, G. Zdunić, M. Bubola, E. Zyprian and I. Pejić (2020). "Genetic diversity, population structure, and parentage analysis of Croatian grapevine germplasm." Genes **11**(7): 737.
